# Bayesian and Classical Inference for the Generalized Log-Logistic Distribution with Applications to Survival Data

**DOI:** 10.1155/2021/5820435

**Published:** 2021-10-11

**Authors:** Abdisalam Hassan Muse, Samuel Mwalili, Oscar Ngesa, Saad J. Almalki, Gamal A. Abd-Elmougod

**Affiliations:** ^1^Department of Mathematics (Statistics Option) Programme, Pan African University, Institute for Basic Science, Technology and Innovation (PAUSTI), Nairobi 6200-00200, Kenya; ^2^Department of Statistics and Actuarial Sciences, Jomo Kenyatta University of Agriculture and Technology (JKUAT), Nairobi 6200-00200, Kenya; ^3^Department of Mathematics and Physical Sciences, Taita Taveta University, Voi 635-80300, Kenya; ^4^Department of Mathematics and Statistics, College of Science, Taif University, P.O. Box 11099, Taif 21944, Saudi Arabia; ^5^Department of Mathematics, Faculty of Science, Damanhour University, Damanhour, Egypt

## Abstract

The generalized log-logistic distribution is especially useful for modelling survival data with variable hazard rate shapes because it extends the log-logistic distribution by adding an extra parameter to the classical distribution, resulting in greater flexibility in analyzing and modelling various data types. We derive the fundamental mathematical and statistical properties of the proposed distribution in this paper. Many well-known lifetime special submodels are included in the proposed distribution, including the Weibull, log-logistic, exponential, and Burr XII distributions. The maximum likelihood method was used to estimate the unknown parameters of the proposed distribution, and a Monte Carlo simulation study was run to assess the estimators' performance. This distribution is significant because it can model both monotone and nonmonotone hazard rate functions, which are quite common in survival and reliability data analysis. Furthermore, the proposed distribution's flexibility and usefulness are demonstrated in a real-world data set and compared to its submodels, the Weibull, log-logistic, and Burr XII distributions, as well as other three-parameter parametric survival distributions, such as the exponentiated Weibull distribution, the three-parameter log-normal distribution, the three-parameter (or the shifted) log-logistic distribution, the three-parameter gamma distribution, and an exponentiated Weibull distribution. The proposed distribution is plausible, according to the goodness-of-fit, log-likelihood, and information criterion values. Finally, for the data set, Bayesian inference and Gibb's sampling performance are used to compute the approximate Bayes estimates as well as the highest posterior density credible intervals, and the convergence diagnostic techniques based on Markov chain Monte Carlo techniques were used.

## 1. Introduction

Applied statisticians use many probability distributions for reliability and survival studies. The distributions could be applied in different fields such as medicine, engineering, economy, industrial and physical fields, and so many other fields. Exponential distributions, generalized exponential distributions, gamma distributions, generalized gamma distributions, extreme value distributions, Weibull distributions, log-logistic distributions, log-normal distributions, Burr XII distributions, and generalized Weibull distributions are among the most frequently used distributions in survival and reliability analysis.

Typically, researchers in reliability and survival analysis are concerned with the development of new probability models. Log-logistic (LL) distribution is one of the parametric distributions that can be used as a life-testing distribution because of the simplicity of its cumulative distribution and survival function which can both be stated in closed form and because it belongs to the Scale-Shape family [1]. LL is one of the right-skewed, heavy-tailed functions that can be used as an alternative to a log-normal distribution. It resembles the log-normal distribution in shape but has heavier tails. Log-logistic distribution is particularly applicable to model nonmonotone (i.e., unimodal) hazard functions.

It is well understood that the log-logistic model is not appropriate for modelling where the failure rate is monotonic when analyzing time-to-event data with parametric models. It is suitable to use an extension of the model which has a monotone hazard function. Departures from the monotonicity of distribution are typically studied in terms of its shape or more specifically in terms of its skewness (also referred to as asymmetry) and kurtosis.

In this study, we focus on a modification of the log-logistic model because it resembles the log-normal distribution in shape but is better suited for the application in the analysis of survival data when dealing with incomplete data, such as censored observations which are common in such data [2]. The presence of incomplete observations causes difficulties when using log-normal or inverse Gaussian models, since the survival functions in these cases are complicated. On the other hand, since the logarithms of small positive numbers are large negative numbers, the log-normal distribution may give undue weight to very short survival times [1]. For the reasons stated above, we will focus on the log-logistic model whose hazard rate exhibits the aforementioned behaviour.

However, due to the log-logistic model's symmetric property, it may be inadequate for cases where the hazard rate is heavily tailed or skewed, as well as for modelling censored survival data [3–5]. In this study, we studied a modification (or generalization) of the log-logistic parametric survival model and referring to this as the generalized log-logistic distribution given in [6]. The generalized log-logistic distribution reflects the structure of the heavy tails and the skewness and it significantly outperformed the log-logistic distribution in general.

In the statistical literature, with the aim of increasing the versatility of the log-logistic distribution in modelling survival time data, different generalized forms of the distribution have recently been proposed, including a new extension of the LL distribution with applications to actuarial data sets [7], alpha power transformed LL distribution [8, 9], transmuted four-parameter generalized LL distribution [10, 11], a new three-parameter LL distribution [12], extended log-logistic distribution [13], exponentiated LL geometric distribution [14], the LL Weibull distribution [15], beta LL distribution [16], McDonald LL distribution [2], transmuted LL distribution [17], Marshal-Olkin LL distribution [18], the Zografos-Balakrishnan LL distribution [19], and exponentiated LL distribution [20]. More details about the modifications and recent generalizations of the log-logistic distribution can be found in [21].

In addition, other authors have studied the Bayesian inference of the LL distribution and some of its generalizations. dos Santos et al. [22] developed a Bayesian analysis of the transmuted LL distribution. Yahaya and Dewu [23] studied the Bayesian estimation of the scale parameter for the LL distribution using Chi-square and Maxwell priors. Abbas and Tang [24] studied the objective Bayesian analysis of the LL distribution using the reference and Jeffreys prior. Al-Shomrani et al. [25] focused on the application of the Markov chain Monte Carlo (McMC) techniques for estimating the unknown parameters of the LL distribution. Guure et al. [26] explored the Bayesian inference of the LL distribution for the interval-censored data. Kang et al. [27] proposed the noninformative priors for the LL distribution. Chaudhary and Kumar [28] studied the Bayesian estimation of the three-parameter exponentiated LL distribution. Akhtar et al. [29] discussed the Bayesian analysis of the LL distribution using the Laplace approximation. Chaudhary [30] proposed the Bayesian analysis of the two-parameter exponentiated LL distribution.

The log-logistic distribution has large-scale applications in analyzing time-to-event data. The model is closed under both proportionality (multiplication) of failure time and proportionality of odds, though it is not a proportional hazard (PH) model. However, regarding this issue, Khan and Khosa [6] presented generalized log-logistic distribution that belongs to the proportional hazard models. The proposed distribution has similar properties to the 2-parameter log-logistic distribution and approaches the Weibull distribution in limit. However, its statistical and mathematical properties, as well as inferential procedures, have not received attention so far. On the other hand, they discussed the classical inference of the proposed distribution under the PH regression framework. However, much work still has to be done. In this paper, we focused on the Bayesian and classical inference of the generalized log-logistic distribution as a generalized distribution, not as a regression model.

Additionally, for the applied cases, especially in the survival modelling, the GLL model could be applicable in the following cases: (1) modelling the “asymmetric monotonically right-skewed” heavy tail data sets; (2) modelling the “bathtub-shaped hazard rate” data sets like data set I; (3) in “survival analysis,” the GLL distribution could be chosen for modelling proportional hazard frameworks; (4) in the medical field, the GLL distribution could be considered in modelling the “bladder cancer data sets” which have “reversed bathtub-shaped HRF” as illustrated in data set I; and (5) in the reliability and survival analysis, the proposed distribution can be an alternative to the Weibull distribution since it can be closed under both accelerated failure time (AFT) and PH models since the Weibull distribution fails to model unimodal data. For these based on ground reasons, we are motivated to study and introduce the GLL distribution.

Thus, the main goal of this research article is to propose and study a generalized log-logistic distribution, which extends the exponential, Weibull, log-logistic, and Burr XII distributions, with the hope that the proposed distribution may have a better fit compared to these distributions and other 3-parametric distributions in certain practical situations. In addition, we would provide a comprehensive account of the mathematical and statistical properties of the proposed model. The proposed model's formulae are simple and tractable, and, with the use of modern computer software and its numerical capabilities, the proposed model could be a great addition to the arsenal of applied mathematicians and statisticians in the areas like medicine, engineering, economics, social sciences, and biology, among others. Finally, we discussed the Bayesian model formulation of the proposed distribution.

The rest of the paper is organized as follows.  Section 2 describes the distribution functions for the GLL distribution, its submodel distributions, and some of its basic properties. Some mathematical properties of the GLL distribution are derived in Section 3.  Section 4 describes the maximum likelihood for the estimation parameters of GLL distribution.  Section 5 discusses the findings of a simulation study that was conducted to estimate and compare the performances of the proposed estimators.  Section 6 presents an analysis of a real-life data set. The Bayesian model formulation for the proposed distribution is discussed in Section 7.  Section 8 presents the Bayesian analysis of a real-life data set using Markov chain Monte Carlo techniques. Finally, Section 9 summarizes the study with some concluding remarks.

## 2. The Generalized Log-Logistic Distribution

The generalized log-logistic distribution is a continuous probability distribution with positive support ℝ on a subset of (0,  *∞*) with three parameters. It is a generalization of the two-parameter log-logistic distribution. The generalization of log-logistic distribution for censored survival data can be traced back to Singh et al. [3] who discussed a generalized log-logistic distribution and applied it to censored survival data and proposed a generalized log-logistic model and introduced the shape parameter and then they used it to fit a lung cancer data. Prentice [31] proposed a generalization for quantile response data and discussed several of its uses.

Since many continuous probability distributions are commonly applied for parametric models in survival analysis like the exponential, Gompertz, Weibull, log-normal, log-logistic, and the gamma distribution, GLL is also applicable for survival data analysis. There are a number of probability functions that are related to continuous probability distributions; we will concentrate on functions that are related to the lifetime distributions as a random variable in this study.

### 2.1. Hazard (Failure) Rate Function

SThe hazard (failure) rate function plays an important role in survival analysis. It is the most popular function for analyzing and modelling lifetime data because of its intuitive interpretation of the amount of risk to fail associated with a unit time *t*, applicable for describing the lifetime distribution of engineered and other components. The hazard rate is more informative than all of the other functions in lifetime distributions. Because of this, the authors in [6] started their work by defining the hazard rate of the GLL distribution. Cox and Oakes [32] described the reason why the hazard rate is considered when we are dealing with the survival data. They gave a number of reasons including the fact that hazard rate-based models are often convenient when there is incomplete information (censoring) or there are several types of failure rates; also hazard rate is a special form of the intensity function, and last but not least the hazard rate function can be derived from all other functions that we use to describe lifetime distributions.

The hazard rate function describes how the instantaneous failure rate changes over time. For the GLL distribution, the hazard rate function plots are given in

(1) $$\begin{array}{c} h\left(x;\;\theta \right)=\;\frac{\alpha k{\left(kx\right)}^{\alpha -1}}{\left[1+{\left(\eta x\right)}^{\alpha }\right]}\;,\;x\geq 0,k,\;\alpha ,\;\eta >0, \end{array}$$

where *k* > 0, *β* > 0, *η* > 0  are parameters and *θ*=(*k*,  *α*,  *η*)′.

**Figure 1:**
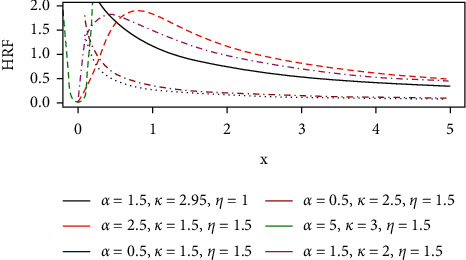
The hazard curve of the GLL distribution.

It can be easily seen from equation (1) that the hazard rate function is monotonically decreasing for *α* ≤ 1 and unimodal when *α* ≤ 1. That is, it initially increases to a maximum at *t*= [(*α* − 1)/*λ*^*α*^]^(1/*α*)^ and then decreases to zero monotonically as *t* ⟶*∞*. The HRF plots are shown in Figure 1.

### 2.2. Submodels

The proposed distribution consists of a number of important submodels that are widely used in parametric survival modelling. These include the log-logistic distribution, the standard log-logistic distribution, the Burr XII distribution, the Weibull distribution, and the exponential distribution. The propositions below relate the GLL to the log-logistic, standard log-logistic, Burr XII, Weibull, and exponential distributions.

#### 2.2.1. Log-Logistic Distribution

**Proposition 1 .**

Let *X* ~ GLL(*α*, *k*, *η*). If *η* depends on *k* via *k*=*η*, then the hazard rate function of (1) reduces to the hazard rate function of the log-logistic distribution.

**Proof**

The hazard rate function of the generalized log-logistic distribution is given by

(2) $$\begin{array}{c} h\left(x;\;\theta \right)=\;\frac{\alpha k{\left(kx\right)}^{\alpha -1}}{\left[1+{\left(\eta x\right)}^{\alpha }\right]}. \end{array}$$

If we replace *η*=*k*, it gives us

(3) $$\begin{array}{c} h\left(x;\;\theta \right)=\;\frac{\alpha k{\left(kx\right)}^{\alpha -1}}{\left[1+{\left(kx\right)}^{\alpha }\right]}\;=\frac{\alpha k{\left(kx\right)}^{\alpha -1}}{\left[1+{\left(kx\right)}^{\alpha }\right]}, \end{array}$$

which is the hazard rate function form of a log-logistic distribution with the two unknown parameters (*k*, *α*). When  *θ*=(*k*, *α*)′, *k*=(1/*β*) is the rate parameter.

It is easy to verify that the hazard rate function of the log-logistic distribution is monotonically decreasing for 0 < *α* ≤ 1 and unimodal for *α* > 1 (decreases and then increases with the maximum at *x*=(1/*k*)(*α* − 1)^(1/*α*)^).

#### 2.2.2. Standard Log-Logistic Distribution

**Proposition 2 .**

Let *X*  ~ GLL(*α*, *k*, *η*). If *η* depends on *k* via *k*=*η*=1, then the hazard rate function of (1) reduces to the hazard rate function of the standard log-logistic distribution.

**Proof**

The hazard rate function of the generalized log-logistic distribution is given by

(4) $$\begin{array}{c} h\left(x;\;\theta \right)=\;\frac{\alpha k{\left(kx\right)}^{\alpha -1}}{\left[1+{\left(\eta x\right)}^{\alpha }\right]}. \end{array}$$

If we replace *η*=*k*=1, it gives us

(5) $$\begin{array}{c} h\left(x;\;\theta \right)=\;\frac{\alpha \cdot 1{\left(1\cdot x\right)}^{\alpha -1}}{\left[1+{\left(1\cdot x\right)}^{\alpha }\right]}\\ =\frac{\alpha {\left(x\right)}^{\alpha -1}}{\left[1+x^{\alpha }\right]}, \end{array}$$

which is the hazard rate function form of a standard log-logistic distribution with one unknown parameter (*α*). Hence, the proof.

It should be noted that *x* > 0, is the distribution‘s support, and *α* is the distribution's shape parameter. It is easy to verify that the hazard rate function of the log-logistic distribution is monotonically decreasing for 0 < *α* ≤ 1 and unimodal for *α* > 1 (decreases and then increases with the maximum at *x*=(*α* − 1)^(1/*α*)^).

#### 2.2.3. Burr XII Distribution

**Proposition 3 .**

Let *X*  ~ GLL(*α*, *k*, *η*). If *η* depends on *k* via *η*=*kλ*^−(1/*α*)^, *λ* > 0, then the hazard rate function of (1) reduces to the hazard rate function of the Burr XII distribution.

**Proof**

The hazard rate function of the generalized log-logistic distribution is given by

(6) $$\begin{array}{c} h\left(x;\;\theta \right)=\;\frac{\alpha k{\left(kx\right)}^{\alpha -1}}{\left[1+{\left(\eta x\right)}^{\alpha }\right]}. \end{array}$$

If we replace *η*=*kλ*^−(1/*α*)^, it gives us

(7) $$\begin{array}{c} h\left(x;\;\theta \right)=\;\frac{\alpha k{\left(kx\right)}^{\alpha -1}}{\left[1+{\left(k\lambda^{-\left(1/\alpha \right)}x\right)}^{\alpha }\right]}\\ =\;\frac{\alpha k{\left(kx\right)}^{\alpha -1}}{\left[1+\left(k\lambda^{-\;\left(\alpha /\alpha \right)}x^{\alpha }\right)\right]}\;=\frac{\alpha kx^{\alpha -1}}{\left[1+x^{\alpha }\right]}, \end{array}$$

which is the hazard rate function form of a Burr XII distribution with two unknown parameters (*α*, *k*). Hence, the proof.

The Burr XII hazard function is monotonically decreasing for *α* ≤ 1 and upside-down bathtub shapes curve for *α* > 1 (which means that it initially increases, attains a maximum at *x*=(*α* − 1)^(1/*α*)^, and then decreases to zero at (*x*⟶*∞*).

#### 2.2.4. Weibull Distribution

**Proposition 4 .**

Let *X*  ~ GLL(*α*, *k*, *η*). If *η*^*α*^⟶0, then the hazard rate function of the GLL (1) approaches the hazard rate function of the Weibull distribution.

**Proof**

If we now let *η*^*α*^⟶0, then, from the hazard rate function of the GLL given by

(8) $$\begin{array}{c} h\left(x;\;\theta \right)=\;\frac{\alpha k{\left(kx\right)}^{\alpha -1}}{\left[1+{\left(\eta x\right)}^{\alpha }\right]}, \end{array}$$

we have that

(9) $$\begin{array}{c} h\left(x;\;\theta \right)=\;\frac{\alpha k{\left(kx\right)}^{\alpha -1}}{\left[1+\left(0\right]\right.}, \end{array}$$

which by simplifying gives

(10) $$\begin{array}{c} h\left(t;\;\theta \right)=\;\alpha k{\left(kx\right)}^{\alpha -1}, \end{array}$$

which is a hazard function of a Weibull distribution with the unknown parameters (*α*, *k*). This property of the GLL enables it to handle monotonically increasing hazard satisfactorily with *α* > 1 and *λ* close to zero (very small).

It is clear from (10) that, for 0 < *α* < 1, the hazard rate function decreases, for *α* > 1, the hazard rate function increases, and for *α*=1, the hazard rate function decreases.

The distribution reduces to exponential for *α*=1.

#### 2.2.5. Exponential Distribution

**Proposition 5 .**

Similarly, if we now let *α*=1, then the hazard rate function of (10) reduces to the hazard rate function of the exponential distribution.

**Proof**

From (10), we have that the hazard rate function is

(11) $$\begin{array}{c} h\left(t;\;\theta \right)=\;\alpha k{\left(kx\right)}^{\alpha -1}, \end{array}$$

and if we replace *α*=1,

(12) $$\begin{array}{c} h\left(t;\;\theta \right)=\;k\cdot 1{\left(1\cdot t\right)}^{1-1}, \end{array}$$

which by simplifying gives

(13) $$\begin{array}{c} h\left(t;\;\theta \right)=\;k, \end{array}$$

which is the hazard rate function of an exponential distribution. This property makes the exponential distribution be inadequate to describe survival data. Hence, the proof.

The summary of the submodels for the proposed distribution is summarized in Table 1.

### 2.3. The Probability Density Function

**Figure 2:**
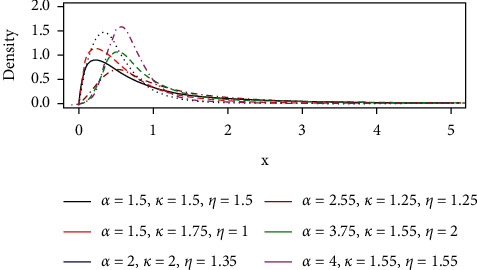
The pdf curve of the GLL distribution.

The pdf of the GLL distribution with three unknown parameters can be obtained by applying the following equation and the pdf plots are shown in Figure 2.

(14) $$\begin{array}{c} f\left(x;\;\theta \right)=h\left(x,\theta \right)\exp\left\{-\int_{0}^{x}h\left(x\right)\text{d}x\right\}. \end{array}$$

Simplifying gives

(15) $$\begin{array}{c} f\left(x;\;\theta \right)=\;\frac{\alpha k{\left(kx\right)}^{\alpha -1}}{{\left[1+{\left(\eta x\right)}^{\alpha }\right]}^{\left(k^{\alpha }/\eta^{\alpha }\right)+1}},\;x\geq 0,k,\;\alpha ,\;\eta >0. \end{array}$$

### 2.4. The Survival (or Reliability) Function

**Figure 3:**
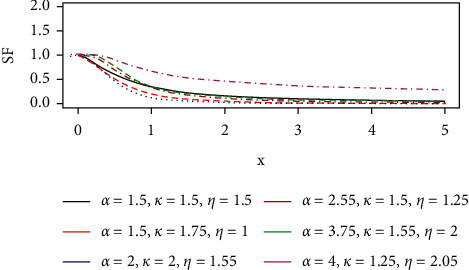
The survival curves of the GLL distribution.

The survival (reliability) function of the GLL distribution that represents the probability that observation does not fail until *t* is given below and its plots are shown in Figure 3.

(16) $$\begin{array}{c} S\left(x;\;\theta \right)=\frac{f\left(x;\;\theta \right)\;}{h\left(x;\;\theta \right)\;}. \end{array}$$

Simplifying gives

(17) $$\begin{array}{c} S\left(x;\;\theta \right)=\;{\left[1+{\left(\eta x\right)}^{\alpha }\right]}^{-\left(k^{\alpha }/\eta^{\alpha }\right)},\;x\geq 0,k,\alpha ,\eta >0. \end{array}$$

### 2.5. Cumulative Distribution Function of the GLL Distribution

**Figure 4:**
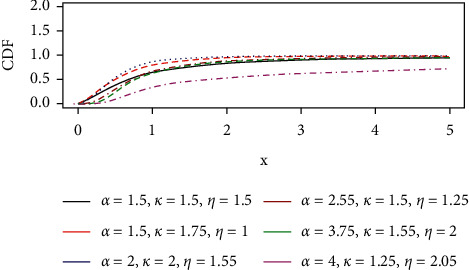
The CDF curve of the GLL distribution.

The cumulative distribution function (CDF), also known as the lifetime distribution function, of the GLL distribution is of the form below and the CDF plots are shown in Figure 4.

(18) $$\begin{array}{c} F\left(x;\;\theta \right)=\frac{{\left[1+{\left(\eta x\right)}^{\alpha }\right]}^{\left(k^{\alpha }/\eta^{\alpha }\right)}-1\;}{{\left[1+{\left(\eta x\right)}^{\alpha }\right]}^{\left(k^{\alpha }/\eta^{\alpha }\right)}},\;x\geq 0,k,\;\alpha ,\;\eta >0, \end{array}$$

where *k* > 0, *β* > 0, *η* > 0  are parameters and *θ*=(*k*,  *α*,  *η*)′.

### 2.6. The Reversed Hazard Rate Function

The reversed hazard rate (also known as the retro hazard) is defined as the ratio of pdf to the corresponding CDF. The retro hazard is written as follows:

(19) $$\begin{array}{c} r\left(x;\;\theta \right)=\frac{f\left(x;\;\theta \right)}{F\left(x;\theta \right)}. \end{array}$$

Reversed hazard rate function plays an important role in the analysis of censored data and in the estimation of the survival function. The following equation gives us the basic relationship between hazard rate function and the reversed hazard rate function.

(20) $$\begin{array}{c} r\left(x;\;\theta \right)=\;\frac{h\left(x;\;\theta \right)S\left(x;\;\theta \right)}{1-S\left(x;\;\theta \right)}. \end{array}$$

The applications of hazard rate function in survival analysis are well known. Recently, the reversed hazard rate function has gained popularity among applied statisticians; for more information, see [33, 34]. Block et al. [33] showed that the hazard rate function plays an essential role in the analysis of right-censored data, while the retro hazard plays an essential role in the analysis of left-censored data.

The reversed hazard rate function of the GLL distribution takes the form

(21) $$\begin{array}{c} r\left(x;\;\theta \right)=\frac{f\left(x;\;\theta \right)}{F\left(x;\;\theta \right)}=\frac{\left(\alpha k{\left(kt\right)}^{\alpha -1}/{\left[1+{\left(\lambda x\right)}^{\alpha }\right]}^{\left(k^{\alpha }/\lambda^{\alpha }\right)+1}\right)}{{\left[1+{\left(\lambda x\right)}^{\alpha }\right]}^{\left(k^{\alpha }/\lambda^{\alpha }\right)}}. \end{array}$$

Simplifying gives

(22) $$\begin{array}{c} r\left(x;\;\theta \right)=\;\frac{\alpha k{\left(kx\right)}^{\alpha -1}}{{\left[1+{\left(\lambda x\right)}^{\alpha }\right]}^{\left(k^{\alpha }/\lambda^{\alpha }\right)+1}-\left[1+{\left(\lambda x\right)}^{\alpha }\right]\;},\;x\geq 0,k,\;\alpha ,\;\eta >0. \end{array}$$

**Figure 5:**
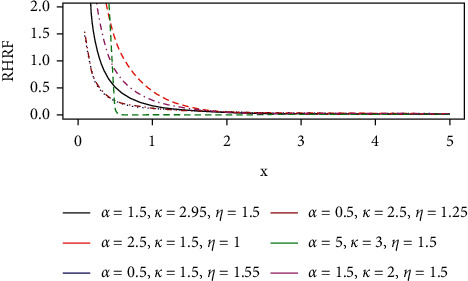
The reversed hazard rate curves of the GLL distribution.

The reversed hazard rate plots are shown in Figure 5.

### 2.7. The Cumulative Hazard Function

The cumulative hazard function of the GLL distribution takes the form

(23) $$\begin{array}{c} H\left(x;\;\theta \right)=\;-\log\;S\left(x;\;\theta \right)=\;\int_{0}^{x}h\left(x;\;\theta \right)\text{d}x. \end{array}$$

Simplifying gives

(24) $$\begin{array}{c} H\left(x;\theta \right)=\;\frac{k^{\alpha }}{\lambda^{\alpha }}\log\;\left[1+{\left(\lambda x\right)}^{\alpha }\right],\;x\geq 0,k,\;\alpha ,\;\eta >0, \end{array}$$

where *k* > 0, *α* > 0, *λ* > 0  are parameters and *θ*=(*k*,  *α*,  *η*)′.

### 2.8. The Hazard Rate Average (FRA) Function

The HRA function of *X* is expressed as

(25) $$\begin{array}{c} \text{HRA}\left(x;\;\theta \right)=\;\frac{H\left(x;\;\theta \right)}{x}=\frac{\int_{\text{0}}^{x}h\left(x;\;\theta \right)\text{d}x}{x},\;x>0, \end{array}$$

where *H*(*x*;  *θ*) is the cumulative hazard function. An analysis of HRA(*x*;  *θ*) on *t* enables us to find increasing hazard rate average and decreasing hazard rate average.

## 3. Some Mathematical Properties of the GLL Distribution

In this section, we present some mathematical properties of the GLL distribution. The functions that we discussed in Section 2 are not the only ways that we can define the GLL distribution, but there are other mathematical functions that we can use to describe the lifetime distributions of a random variable *X*. These include quantile function and its related results, moments and its related properties, *r*^th^ central moments, residual life and reversed residual life functions, and other mathematical properties.

### 3.1. The Quantile Function and Related Results

The quantile function (which is the inverse of the CDF) is crucial in statistical and quantitative data analysis. A probability distribution can be defined in terms of either the quantile function or the cumulative distribution function [35]. The quantiles of the proposed distribution with various parameter values are given in Table 2.

**Theorem 1 .**

If *T*  ~ GLL(*k*,  *α*,  *η*), then the quantile function, lower quartile, median, and the upper quartile of the GLL distribution, respectively, are given by

(26) $$\begin{array}{c} X_{q}=F^{-1}\left(q;k,\alpha ,\;\eta \right)=\frac{{\left\{{\left[\;1/\left(1-p\right)\right]}^{\left(\eta^{\alpha }/k^{\alpha }\right)}-1\right\}}^{\left(1/\alpha \right)}}{\eta }, \end{array}$$

(27) $$\begin{array}{c} X_{q_{1}}=\frac{{\left\{{\left[4/3\right]}^{\left(\eta^{\alpha }/k^{\alpha }\right)}-1\right\}}^{\left(1/\alpha \right)}}{\eta }, \end{array}$$

(28) $$\begin{array}{c} X_{q_{2}}=\text{Median}=\;\frac{{\left\{2^{\left(\eta^{\alpha }/k^{\alpha }\right)}-1\right\}}^{\left(1/\alpha \right)}}{\eta }, \end{array}$$

(29) $$\begin{array}{c} X_{q_{3}}=\frac{{\left\{4^{\left(\eta^{\alpha }/k^{\alpha }\right)}-1\right\}}^{\left(1/\alpha \right)}}{\eta }. \end{array}$$

**Proof**

The quantile function of GLL distribution is derived by finding the value of *Q* for which

(30) $$\begin{array}{c} 1-{\left[1+{\left(\eta x\right)}^{\alpha }\right]}^{-\left(k^{\alpha }/\eta^{\alpha }\right)}=p,\\ X_{q\;}=F^{-1}\left(q;k,\alpha ,\;\eta \right)=\;{\left[1+{\left(\eta q\right)}^{\alpha }\right]}^{-\left(k^{\alpha }/\eta^{\alpha }\right)}=1-p\\ =\;\frac{1}{{\left[1+{\left(\eta q\right)}^{\alpha }\right]}^{\left(k^{\alpha }/\eta^{\alpha }\right)}}=1-p\\ =\;{\left[1+{\left(\eta q\right)}^{\alpha }\right]}^{\left(k^{\alpha }/\eta^{\alpha }\right)}=\frac{1}{1-p}\\ =1+{\left(\eta q\right)}^{\alpha }={\left(\frac{1}{1-p}\right)}^{\left(\eta^{\alpha }/k^{\alpha }\right)}\\ =\;{\left(\eta q\right)}^{\alpha }={\left(\frac{1}{1-p}\right)}^{\left(\eta^{\alpha }/k^{\alpha }\right)}-1\\ =\eta q=\;{\left\{{\left[\;\frac{1}{1-p}\right]}^{\left(\eta^{\alpha }/k^{\alpha }\right)}-1\right\}}^{\left(1/\alpha \right)}\;,\\ ∴q=\frac{{\left\{{\left[\;1/\left(1-p\right)\right]}^{\left(\eta^{\alpha }/k^{\alpha }\right)}-1\right\}}^{\left(1/\alpha \right)}}{\eta }\;, \end{array}$$

where *p*  ∈ [0,1). *k* > 0, *α* > 0, *η* > 0. Hence the proof.

Similarly, we can prove (27)–(29) by applying the following values: the lower quartile = 1/4, median = 2/4 = 1/2, and the upper quartile = 3/4.

Lower quartile is

(31) $$\begin{array}{c} X_{q_{1}}=\frac{{\left\{{\left[\;4/3\right]}^{\left(\eta^{\alpha }/k^{\alpha }\right)}-1\right\}}^{\left(1/\alpha \right)}}{\eta }. \end{array}$$

Median is

(32) $$\begin{array}{c} X_{q_{2}}=\text{median}=\;\frac{{\left\{2^{\left(\eta^{\alpha }/k^{\alpha }\right)}-1\right\}}^{\left(1/\alpha \right)}}{\eta }. \end{array}$$

Upper quartile is

(33) $$\begin{array}{c} X_{q_{3}}=\frac{{\left\{4^{\left(\eta^{\alpha }/k^{\alpha }\right)}-1\right\}}^{\left(1/\alpha \right)}}{\eta }. \end{array}$$

#### 3.1.1. Skewness and Kurtosis

The following relationship defines the mathematical form of the Galton Skewness and Moors Kurtosis of the GLL model with three parameters:

(34) $$\begin{array}{c} S_{K}=\;\frac{Q\left(3/4\right)\;+Q\left(1/4\right)\;-2Q\left(2/4\right)}{Q\left(3/4\right)-Q\left(1/4\right)},\\ K_{M}=\;\frac{Q\left(7/8\right)\;+Q\left(3/8\right)\;-Q\left(5/8\right)-Q\left(1/8\right)}{Q\left(6/8\right)-Q\left(2/8\right)}, \end{array}$$

where *Q* describes different quartile values.

The above equations can be determined as functions of the GLL quantile function. The advantages of these measures are that they are less sensitive in the presence of outliers and that they exist even when the distribution is lacking moments.

### 3.2. The Random Deviate Generation Functions

Let U be a random variable with a uniform distribution (0,1) and an inverse CDF, *F*(.). Then any sample drawn from *F*^−1^(*u*) is assumed to have been drawn from *F*(.). As a result, using GLL (*k*,  *α*,  *η*), the random deviate can be generated as follows:

(35) $$\begin{array}{c} x=\;\frac{{\left\{{\left[1/\left(1-u\right)-1\right]}^{\left(\lambda^{\alpha }/k^{\alpha }\right)}\right\}}^{\left(1/\alpha \right)}}{\lambda },\;0<u<1, \end{array}$$

where *u* follows *U*(0,1) distribution.

### 3.3. The *r*^th^ Moments and Related Results

Numerous important characteristics and properties of a probability distribution such as mean, variance, kurtosis, and skewness can be obtained from its moments. Moments are extremely important and play a central role in statistical analysis, especially in applications. The important moment functions, such as the moments, *r*^th^ moment, *r*^th^ central moment, mean, variance, skewness, and kurtosis of the proposed distribution, are presented.

**Theorem 2 .**

If *T*  ~ GLL (*k*,  *α*,  *η*), then the *r*^th^ power, negative moments, and logarithmic moments are given, respectively, by

(36) $$\begin{array}{c} E\left(T^{r}\right)=\frac{k^{\alpha }}{\eta^{\alpha +r}}\frac{\Gamma \left(\left(k^{\alpha }/\eta^{\alpha }\right)-\left(r/\alpha \right)\right)\Gamma \left(\left(r/\alpha \right)+1\right)}{\Gamma \left(\left(k^{\alpha }/\eta^{\alpha }\right)+1\right)}\;,\;\text{for }\frac{\alpha k^{\alpha }}{\;\eta^{\alpha }}>r, \end{array}$$

(37) $$\begin{array}{c} E\left(T^{-r}\right)=\frac{\lambda^{\alpha +r}}{k^{\alpha }}\frac{\Gamma \left(\left(k^{\alpha }/\eta^{\alpha }\right)+1\right)}{\Gamma \left(\left(k^{\alpha }/\eta^{\alpha }\right)-\left(r/\alpha \right)\right)\Gamma \left(\left(r/\alpha \right)+1\right)}. \end{array}$$

**Proof**

We have

(38) $$\begin{array}{c} E\left(T^{r}\right)=\int_{0}^{\infty }t^{r}f\left(t;k,\alpha ,\;\eta \right)\text{d}t=\int_{0}^{\infty }t^{r}\frac{\alpha k{\left(kt\right)}^{\alpha -1}}{{\left[1+{\left(\;\eta t\right)}^{\alpha }\right]}^{\left(k^{\alpha }/\eta^{\beta }\right)+1}}\text{ d}t\;=\;\frac{\alpha k}{\Gamma \left(\left(k^{\alpha }/\eta^{\alpha }\right)+1\right)}\int_{0}^{\infty }t^{r}\frac{{\left(kt\right)}^{\alpha -1}}{1+{\left(\;\eta t\right)}^{\alpha }}\text{d}t\\ =\frac{k^{\alpha }}{\eta^{\alpha +r}}\frac{\Gamma \left(\left(k^{\alpha }/\eta^{\alpha }\right)-\left(r/\alpha \right)\right)\Gamma \left(\left(r/\alpha \right)+1\right)}{\Gamma \left(\left(k^{\alpha }/\eta^{\alpha }\right)+1\right)},\;\text{for }\frac{\alpha k^{\alpha }}{\;\eta^{\alpha }}>r. \end{array}$$

Similarly, we can prove (37).

#### 3.3.1. Mean and Variance

**Corollary 1 .**

If *T*  ~ GLL (*k*,  *α*,  *η*), then the mean and variance are given, respectively, as follows.

**The mean** of the GLL distribution is

(39) $$\begin{array}{c} \mu =E\left(T\right)=\frac{k^{\alpha }}{\eta^{\alpha }}\frac{\Gamma \left(\left(k^{\alpha }/\eta^{\alpha }\right)-\left(1/\alpha \right)\right)\Gamma \left(\left(1/\alpha \right)+1\right)}{\Gamma \left(\left(k^{\alpha }/\eta^{\alpha }\right)+1\right)}. \end{array}$$

This is provided that (*αk*^*α*^/*η*^*α*^) > 1.

**The Variance** of the GLL distribution is

(40) $$\begin{array}{c} \sigma^{2}=V\left(T\right)=E\left(T^{2}\right)-{\left(E\left(T\right)\right)}^{2}\\ =\frac{k^{\;\alpha }}{\eta^{\;\alpha +2}}\frac{\Gamma \left(\left(k^{\alpha }/\eta^{\alpha }\right)-\left(2/\alpha \right)\right)\Gamma \left(\left(2/\alpha \right)+1\right)}{\Gamma \left(\left(k^{\alpha }/\eta^{\alpha }\right)+1\right)}-\;{\left(\frac{k^{\;\alpha }}{\eta^{\;\alpha }}\frac{\Gamma \left(\left(k^{\alpha }/\eta^{\alpha }\right)-\left(1/\alpha \right)\right)\Gamma \left(\left(1/\alpha \right)+1\right)}{\Gamma \left(\left(k^{\alpha }/\eta^{\alpha }\right)+1\right)}\right)}^{2}. \end{array}$$

This is provided that (*αk*^*α*^/*η*^*α*^) > 2.

### 3.4. The *r*^th^ Central Moments

**Corollary 2 .**

If *T*  ~ GLL (*k*,  *α*,  *η*), then the cumulants of the first, second, and *r*^*t*h^ central moments, are given, respectively, by

(41) $$\begin{array}{c} c_{1}=\mu_{1}^{\prime }=E\left(T\right)=\frac{k^{\;\alpha }}{\eta^{\;\alpha }}\frac{\Gamma \left(\left(k^{\alpha }/\eta^{\alpha }\right)-\left(1/\alpha \right)\right)\Gamma \left(\left(1/\alpha \right)+1\right)}{\Gamma \left(\left(k^{\alpha }/\eta^{\alpha }\right)+1\right)},\\ c_{2}=\mu_{2}^{\prime }-\mu_{1}^{2\prime }=E\left(T^{2}\right)-{\left(E\left(T\right)\right)}^{2}\;=\;\frac{k^{\;\alpha }}{\eta^{\;\alpha +2}}\frac{\Gamma \left(\left(k^{\alpha }/\eta^{\alpha }\right)-\left(2/\alpha \right)\right)\Gamma \left(\left(2/\alpha \right)+1\right)}{\Gamma \left(\left(k^{\alpha }/\eta^{\alpha }\right)+1\right)}-\;{\left(\frac{k^{\beta }}{\eta^{\;\alpha }}\frac{\Gamma \left(\left(k^{\alpha }/\eta^{\alpha }\right)-\left(1/\alpha \right)\right)\Gamma \left(\left(1/\alpha \right)+1\right)}{\Gamma \left(\left(k^{\alpha }/\eta^{\alpha }\right)+1\right)}\right)}^{2},\\ c_{r}=\;\mu_{r}^{\prime }-\;\overset{r-1}{\underset{n=1}{\sum }}\left(\begin{array}{c} r-1\\ n-1 \end{array}\right)c_{n}\;\mu_{r-m}^{\prime }=\;\frac{k^{\;\alpha }}{\eta^{\;\alpha +r}}\frac{\Gamma \left(\left(k^{\alpha }/\eta^{\alpha }\right)-\left(r/\alpha \right)\right)\Gamma \left(\left(r/\alpha \right)+1\right)}{\Gamma \left(\left(k^{\alpha }/\eta^{\alpha }\right)+1\right)}\\ -\overset{r-1}{\underset{n=1}{\sum }}\left(\begin{array}{c} r-1\\ n-1 \end{array}\right)c_{n}\frac{k^{\;\alpha }}{\eta^{\alpha +\left(r-n\right)}}\frac{\Gamma \left(\left(k^{\alpha }/\eta^{\alpha }\right)-\left(\left(r-n\right)/\alpha \right)\right)\Gamma \left(\left(\left(r-n\right)/\alpha \right)+1\right)}{\Gamma \left(\left(k^{\alpha }/\eta^{\alpha }\right)+1\right)}\;. \end{array}$$

Hence, from Corollary 2, we can derive the skewness and kurtosis of the GLL distribution by computing, respectively:

(42) $$\begin{array}{c} \text{Skewness}=\frac{c_{3}}{{\left(\sigma^{2}\right)}^{\left(3/2\right)}},\\ \text{Kurtosis}=\frac{c_{4}}{{\left(\sigma^{2}\right)}^{2}}. \end{array}$$

### 3.5. Residual and Reverse Residual Life

The residual life has broader applications in survival analysis and risk management. The residual lifetime of the GLL random variable is calculated as follows:

(43) $$\begin{array}{c} R_{\left(t\right)\;}\left(x\right)=\;\frac{S\left(x+t\right)}{S\left(t\right)},\\ R_{\left(t\right)\;}\left(x\right)=\frac{{\left[1+{\left(\eta \left(x+t\right)\right)}^{\alpha }\right]}^{-\left(k^{\alpha }/\eta^{\alpha }\right)}\;}{{\left[1+{\left(\eta t\right)}^{\alpha }\right]}^{-\left(k^{\alpha }/\eta^{\alpha }\right)}}. \end{array}$$

In addition, the reverse residual life of the generalized log-logistic random variable can be calculated as follows:

(44) $$\begin{array}{c} {\hat{R}}_{\left(t\right)\;}\left(x\right)=\;\frac{S\left(x-t\right)}{S\left(t\right)},\\ {\hat{R}}_{\left(t\right)\;}\left(x\right)=\frac{{\left[1+{\left(\eta \left(x-t\right)\right)}^{\alpha }\right]}^{-\left(k^{\alpha }/\eta^{\alpha }\right)}\;}{{\left[1+{\left(\eta t\right)}^{\alpha }\right]}^{-\left(k^{\alpha }/\eta^{\alpha }\right)}}. \end{array}$$

From Table 3, the GLL distribution is clearly numerically versatile in terms of means and variance. Furthermore, the values of CS show that it can be right-skewed, nearly symmetrical, or slightly left-skewed. The CK values show that the GLL distribution can be mesokurtic, leptokurtic, or platykurtic. All of these characteristics demonstrate the GLL distribution flexibility, which remains appealing for modelling purposes.

**Figure 6:**
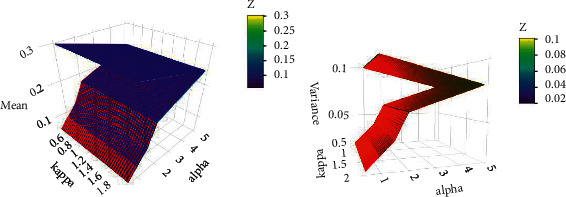
The mean and variance plot for several combinations of alpha and kappa parameters.

**Figure 7:**
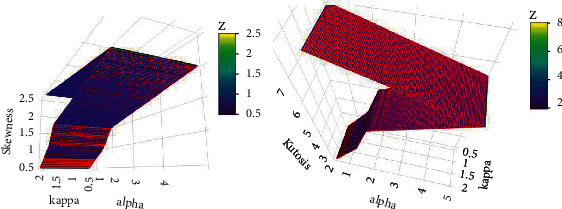
The skewness and kurtosis plot for several combinations of alpha and kappa.

The mean and variance plots for different values of alpha and kappa parameters are shown in Figure 6, while the skewness and kurtosis plots are shown in Figure 7.

## 4. Maximum Likelihood Estimation (MLE)

In this section, the unknown parameters of the generalized log-logistic distribution based on a complete sample are estimated using the maximum likelihood method. Let *X*_1_, *X*_2_,   …,  *X*_*n*_ indicate a random sample of the complete GLL data, and then the sample's likelihood function is given as

(45) $$\begin{array}{c} L=\prod_{i=1}^{n}f\left(x_{i},\;\alpha ,k,\eta \right)\;,\\ L\left(x;\alpha ,\;k,\;\eta \right)=\;\overset{n}{\underset{i=1}{\prod }}\frac{\alpha k{\left(kx_{i}\right)}^{\alpha -1}}{{\left[1+{\left(\eta x_{i}\right)}^{\alpha }\right]}^{\left(k^{\alpha }/\eta^{\alpha }\right)+1}}. \end{array}$$

The log-likelihood function may be expressed as

(46) $$\begin{array}{c} \ell =n\;\log\left(\alpha k\right)+\left(\alpha -1\right)\overset{n}{\underset{i=1}{\sum }}\log\left(kx_{i}\right)-\overset{n}{\underset{i=1}{\sum }}\log\left[1+{\left(\eta x_{i}\right)}^{\alpha }\right]-\left(\frac{k}{\eta }\right)\overset{n}{\underset{i=1}{\sum }}\log\left[1+{\left(\eta x_{i}\right)}^{\alpha }\right]. \end{array}$$

By taking the first derivatives of the log-likelihood function in equation (48) with respect to *α*, *k*,  and *η* and fixing the outcome to zero, we have

(47) $$\begin{array}{c} \frac{\partial \ell }{\partial \alpha }=\frac{n}{\alpha }+\overset{n}{\underset{i=1}{\sum }}\text{log}\left(kx_{i}\right)-\overset{n}{\underset{i=1}{\sum }}\left\{\frac{\left({\left(\eta x_{i}\right)}^{\alpha }\log\left(\eta x_{i}\right)\right)}{\left[1+{\left(\eta x_{i}\right)}^{\alpha }\right]}\right\}-\left(\frac{k}{\eta }\right)\overset{n}{\underset{i=1}{\sum }}\left\{\frac{\left({\left(\eta x_{i}\right)}^{\alpha }\log\left(\eta x_{i}\right)\right)}{\left[1+{\left(\eta x_{i}\right)}^{\alpha }\right]}\right\}, \end{array}$$

(48) $$\begin{array}{c} \frac{\partial \ell }{\partial k}=\frac{n}{k}+nk\left(\alpha -1\right)-\frac{1}{\eta }\overset{n}{\underset{i=1}{\sum }}\text{log}\left(1+\eta x_{i}\right), \end{array}$$

(49) $$\begin{array}{c} \frac{\partial \ell }{\partial \eta }=-\overset{n}{\underset{i=1}{\sum }}\left\{\frac{\left({\left(\eta x_{i}\right)}^{\alpha }\log\left(\eta x_{i}\right)\right)}{\left[1+{\left(\eta x_{i}\right)}^{\alpha }\right]}\right\}-\frac{k}{\eta^{2}}\overset{n}{\underset{i=1}{\sum }}\left\{\frac{\left({\left(\eta x_{i}\right)}^{\alpha }\log\left(\eta x_{i}\right)\right)}{\left[1+{\left(\eta x_{i}\right)}^{\alpha }\right]}\right\}. \end{array}$$

It is worth noting that the MLEs $\hat{\alpha },\hat{k}\text{ and }\hat{\eta }$ of *α*, *k*,  and *η*, respectively, can be obtained by equating the results to zero and numerically solving the system of nonlinear equations. Because the expected information matrix is complicated, the observed information matrix *J*(*θ*) is used to construct confidence intervals for the model parameters. The observed information matrix is given by

(50) $$\begin{array}{c} J\left(\theta \right)=-\;\left[\begin{array}{ccc} \frac{\partial^{2}\ell }{\partial^{2}\alpha } & \frac{\partial^{2}\ell }{\partial \alpha \;\partial k} & \frac{\partial^{2}\ell }{\partial \alpha \;\partial \eta }\\ \; & \frac{\partial^{2}\ell }{\partial^{2}k} & \frac{\partial^{2}\ell }{\partial k\;\partial \partial }\\ \; & \; & \frac{\partial^{2}\ell }{\partial^{2}\eta } \end{array}\right], \end{array}$$

where *θ*=(*α*, *k*,  *η*)′. When the usual regularity conditions are met and the parameters are within the parameter space's interior but not on the boundary, $\sqrt{n}\left(≅\theta -\theta \right)$ converges in distribution to *N*_3_(0, *I*^−1^(*θ*)), where *I*(*θ*) is the expected information matrix. When *I*(*θ*) is replaced by the observed information matrix evaluated at *J*(*θ*), the asymptotic behaviour remains valid. The asymptotic multivariate normal distribution *N*_3_(0, *J*^−1^(*θ*)) can be used to generate 100(1 − *τ*)% two-sided confidence intervals for the model parameters, where *τ* is the significant level.

## 5. Monte Carlo Simulation Study

In this section, we assess the performance of the MLEs estimators for a finite sample of size *n* using a Monte Carlo simulation study. The simulation study based on the generalized log-logistic distribution is carried out to examine the average biases (ABs), the mean square errors (MSEs), the root mean square errors (RMSEs), and maximum likelihood estimates (MLEs) for the model parameters *α*, *k*, and *η*. The simulation experiment was carried out using a variety of simulations with varying sample sizes and parameter values. To generate random samples for the GLL, the quantile function is given in equation [26]. The simulation study was repeated 1500 times, each with sample sizes *n*=50,100,   …,  1500, and the following parameter scenarios in set I: *α*=0.9, *k*=0.5and *η*=2.5, and the following parameter scenarios in set II: *α*=0.8, *k*=0.4 and *η*=2.0.

The MLEs of the GLL model are determined via the nlminb () R-function with the argument method = “BFGS”; see supplementary materials (available here). For each piece of simulated data, say, ($\hat{\alpha },\hat{k},\hat{\eta }$) for *i*=1,2,…,  1000, the AB, RMSE, and MSE of the parameters were computed by

(51) $$\begin{array}{c} \text{AB}=\;\frac{1}{N}\overset{N}{\underset{i=1}{\sum }}\left(\hat{\theta }-\theta \right),\\ \text{MSE}=\;\frac{1}{N}\overset{N}{\underset{i=1}{\sum }}{\left(\hat{\theta }-\theta \right)}^{2},\\ \text{RMSE}=\;\sqrt{\frac{1}{N}\overset{N}{\underset{i=1}{\sum }}{\left(\hat{\theta }-\theta \right)}^{2}}, \end{array}$$

where *θ*=*α*, *k* and *η*.

**Figure 8:**
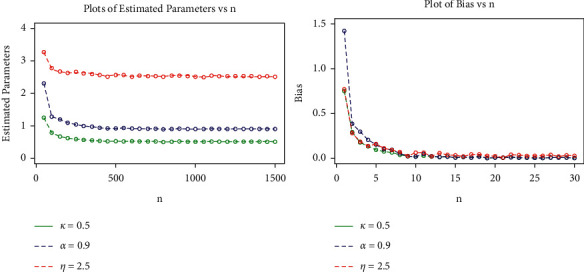
Plots for MLEs and biases of the GLL model for set I of the table.

**Figure 9:**
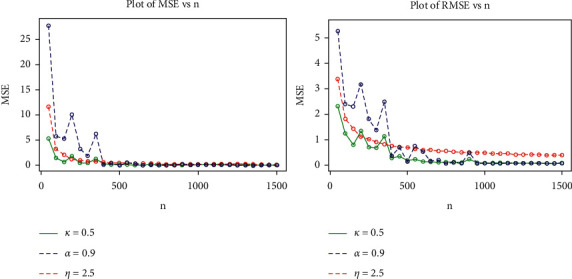
Plots for MSEs and RMSEs of the GLL distribution for the values of set I in the table.

**Figure 10:**
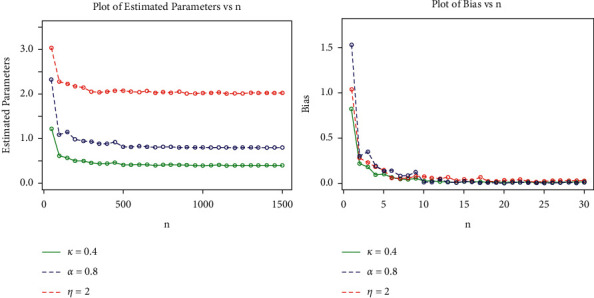
Plots for MLEs and biases of the GLL distribution for the values of set II in the table.

**Figure 11:**
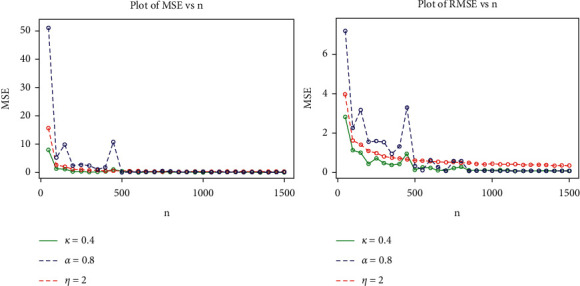
Plots for MSEs and RMSEs of the GLL distribution for the values of set II in the table.

The MLE, AB, and RMSE values of the parameters *α*, *k* and *η* are displayed from various sample sizes. Based on these findings, we conclude that the MLEs perform quite well in estimating the model parameters and that the estimates are fairly stable and are nearer to the true values for these sample sizes.  Table 4 and Figures 8–11 show that as the sample size increases, the MSE and RMSE decrease as expected. Furthermore, as the sample size increases, the AB decreases. In addition, the MLEs of the parameters of the model are very close to the true value. As a result, the maximum likelihood estimates and their asymptotic results can be applied to construct confidence intervals for the model parameters even for a small sample size.

## 6. Data Analysis

In this section, the proposed distribution is fully applied to real-world data set which is taken from literature to demonstrate the ability of the new model. We compare the proposed distribution with the other three parametric survival distributions including gamma, log-normal, log-logistic, exponentiated Weibull, and the Weibull distribution. Also, we have compared the GLL distribution with some of its submodels with two-parameter distribution, namely, Weibull, log-logistic, and the Burr XII distributions.

The density functions of the fitted models are as follows.

(1) Weibull distribution:
  (52) $$\begin{array}{c} f\left(t\right)=\alpha k{\left(kt\right)}^{\alpha -1}\exp\left\{-{\left(kt\right)}^{\alpha }\right\}. \end{array}$$
(2) Log-logistic distribution:
  (53) $$\begin{array}{c} f\left(t\right)=\frac{\alpha k{\left(kt\right)}^{\alpha -1}}{{\left[1+{\left(kt\right)}^{\alpha }\right]}^{2}}. \end{array}$$
(3) Burr XII distribution:
  (54) $$\begin{array}{c} f\left(t\right)=\frac{\alpha kt^{\alpha -1}}{{\left[1+t^{\alpha }\right]}^{-k-1}}. \end{array}$$
(4) Exponentiated Weibull distribution:
  (55) $$\begin{array}{c} f\left(t\right)=\alpha k\lambda {\left(kt\right)}^{\alpha -1}{\left(1-\text{exp}\left\{-{\left(kt\right)}^{\alpha }\right\}\right)}^{\lambda -1}\exp\left\{-{\left(kt\right)}^{\alpha }\right\}. \end{array}$$
(5) Three-parameter log-logistic distribution (or shifted log-logistic distribution):
  (56) $$\begin{array}{c} f\left(t\right)=\;\frac{\alpha /\beta {\left(\left(t-\mu \right)/\beta \right)}^{\alpha -1}}{{\left[1+{\left(\left(t-\mu \right)/k\beta \right)}^{\alpha }\right]}^{2}}. \end{array}$$
(6) Three-parameter log-normal distribution:
  (57) $$\begin{array}{c} f\left(t\right)=\;\frac{\alpha }{\beta }{\left(\frac{t-\mu }{\beta }\right)}^{\alpha -1}\exp\left\{-{\left(\frac{t-\mu }{k\beta }\right)}^{\alpha }\right\}. \end{array}$$
(7) Three-parameter Weibull distribution:
  (58) $$\begin{array}{c} f\left(t\right)=\;\frac{\exp\left\{-\left(1/2\right){\left(\left(\log\left(t-\mu \right)-\alpha \right)/\beta \right)}^{2}\right\}}{\sqrt{2\pi }\beta \left(x-\mu \right)}. \end{array}$$
(8) Three-parameter Gamma distribution:
  (59) $$\begin{array}{c} f\left(t\right)=\;\frac{{\left(t-\mu \right)}^{\alpha -1}\exp-\left(\left(t-\mu \right)/\beta \right)}{\beta^{\alpha }\Gamma \left(\alpha \right)}, \end{array}$$
where *t* > *μ*.

Certain analytical measures are taken into account in order to determine which distribution best fits the applied data. These analytical measures include four discrimination measures: AIC (Akaike Information Criterion), CAIC (Consistent Akaike Information Criterion), BIC (Bayesian Information Criterion), and HQIC (Hannan-Quin Information Criterion). In addition, there are two goodness-of-fit tests: Anderson–Darling (*A*^*∗*^) and Cramer-von Mises (*W*^*∗*^).

The AIC is

(60) $$\begin{array}{c} \text{AIC}=2k-2l. \end{array}$$

The BIC is

(61) $$\begin{array}{c} \text{BIC}=k\;\ln\left(n\right)-2l. \end{array}$$

The CAIC is

(62) $$\begin{array}{c} \text{CAIC}=\frac{2nk}{n-k-1}-2l. \end{array}$$

The HQIC is

(63) $$\begin{array}{c} \text{HQIC}=2k\;\ln\left(\ln\left(n\right)\right)-2l, \end{array}$$

where *l* represents the log-likelihood function evaluated as the MLEs, *n* denotes the sample size, and *k*  denotes the number of model parameters. The goodness-of-fit measures under consideration are as follows.

The Anderson–Darling (*A*^*∗*^) test statistic is given by

(64) $$\begin{array}{c} A^{*}=-n-\frac{1}{n}\overset{n}{\underset{i=1}{\sum }}\left(2l-1\right)\times \left[\ln\;G\left(X_{i}\right)+\ln\left\{1-G\left(X_{n-i+1}\right)\right\}\right]. \end{array}$$

The Cramer-von Mises (*W*^*∗*^) test statistic is given by

(65) $$\begin{array}{c} W^{*}=\frac{1}{12n}+\overset{n}{\underset{i=1}{\sum }}{\left[\frac{2i-1}{2n}+G\left(X_{i}\right)\right]}^{2}, \end{array}$$

where *x*_*i*_ is the *i*th observation in the sample and *n* is the sample size; *x*_*i*_ is calculated when the data is sorted in ascending order.

The best model is the one with the lowest AIC, BIC, CAIC, and HQIC, as well as the *A*^*∗*^, *W*^*∗*^, and K-S tests. Moreover, the best model is also chosen as the one having the highest value of the log-likelihood function, and *p* values for the K-S statistics are also used to compare the competitive models.

### 6.1. Likelihood Ratio Test for Submodels

The GLL distribution has five submodels, namely, log-logistic distribution, Weibull distribution, Burr XII distribution, exponential distribution, and the standard log-logistic distribution. Hence, we have employed the likelihood ratio criterion to test the following hypotheses:

1. *H*_0_ : *η*^*α*^⟶0; that is, the sample is from Weibull distribution. $\;H_{1}:\eta^{\alpha }\underset{\text{not}}{⟶}0$; that is, the sample is GLL
2. *H*_0_ : *η*=*k*; that is, the sample is from log-logistic distribution. *H*_1_ : *η* ≠ *k*; that is, the sample is GLL
3. *H*_0_ : *kλ*^−(1/*α*)^, *λ* > 0; that is, the sample is from Burr XII distribution. *H*_1_ : *kλ*^−(1/*α*)^, *λ* ≤ 0; that is, the sample is GLL
4. *H*_0_ : *η*=*k*=1; that is, the sample is from the standard log-logistic distribution. *H*_1_ : *η* ≠ 1, *k* ≠ 1; that is, the sample is GLL
5. *H*_0_ : *η*=0&*α*=1; that is, the sample is from an exponential distribution. *H*_1_ : *η* ≠ 0&*α* ≠ 1; that is, the sample is GLL

The likelihood ratio test (LRT) is given by

(66) $$\begin{array}{c} \text{LR}=-2\;\ln\frac{\left(L\left({\hat{\theta }}^{*};x\right)\right)}{\left(L\left(\hat{\theta };x\right)\right)}, \end{array}$$

where ${\hat{\theta }}^{*}$ represents the restricted Maximum likelihood estimates under the null hypothesis *H*_0_ and $\hat{\theta }$ represents the unrestricted Maximum likelihood estimates under the alternative hypothesis *H*_1_. Under the null hypothesis, the LRT follows Chi-square distribution with degrees of freedom (df) (d*f*_alt_ − d*f*_null_). If the *p* value is less than 0.05, the null hypothesis is rejected.

### 6.2. An Application to Bladder Cancer Data Set

The following real-world data set is used to demonstrate the proposed methodology. The data in Table 5 below show the remission times (in months) of a sample of 128 bladder cancer patients. The data set is available in [36]. The descriptive statistics for the data set are shown in Table 6 and the likelihood ratio test statistics for the data set are given in Table 7.

For data set I, the asymptotic variance-covariance matrix for the estimated GLL parameters is given by

(67) $$\begin{array}{c} J^{-1}=\;\left[\begin{array}{ccc} 3.0929\times 10^{-4} & 1.7255\times 10^{-3} & 5.8513\times 10^{-4}\\ 1.7255\times 10^{-3} & 3.1612\times 10^{-2} & 5.9347\times 10^{-3}\\ 5.8513\times 10^{-4} & 5.9347\times 10^{-3} & 1.5958\times 10^{-3} \end{array}\right]. \end{array}$$

The information criterion values in Table 8 and the goodness-of-fit tests in Table 9 both demonstrate the superiority of the proposed model over the other competing models.

**Figure 12:**
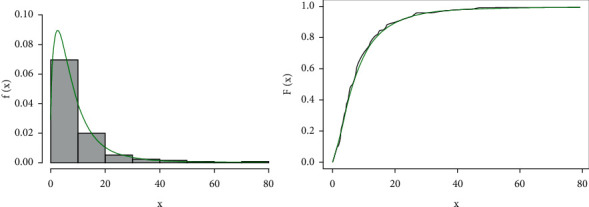
Estimated pdf and CDF of the GLL distribution corresponding to data set I.

**Figure 13:**
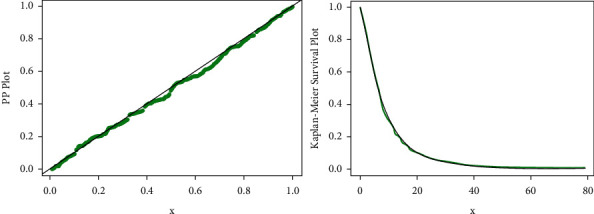
PP and Kaplan–Meier plots of the GLL distribution corresponding to data set I.

The estimated pdf and CDF of the proposed distribution corresponding to the real-world data set are shown in Figure 12 and the Kaplan–Meier and PP plots for the proposed distribution are shown in Figure 13.

#### 6.2.1. TTT Plot

The total time test (TTT) plot plays a central role in determining the best model to fit the given data in terms of the hazard rates. This plot depicts the various forms of the hazard rate. A straight line on the TTT plot indicates that the given data has a constant hazard rate. If the plot is convex, the hazard rate will be decreased; if it is concave, the hazard rates will be increased. The plot for the bathtub shape is first convex and then concave. Similarly, if the hazard rate has an inverted bathtub shape, it will increase first (or concave) and then decrease (or convex). The TTT plot is calculated by using the following formula:

(68) $$\begin{array}{c} G\left(\frac{r}{n}\right)=\;\frac{\sum_{i=1}^{r}x_{i:n}+\left(n-r\right)x_{i:n}}{\sum_{i=1}^{r}x_{i:n}},\;r=x_{i:n}=1,2,\ldots ,n, \end{array}$$

where *x*_*i*:*n*_ are the order statistics.

**Figure 14:**
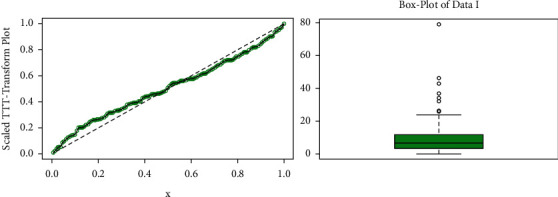
TTT and box plots of data set I.

The TTT and box plots of the data set are presented in Figure 14. These plots indicate that the empirical hazard rate function of the 1st data set is bathtub shape, monotonically increasing.

**Figure 15:**
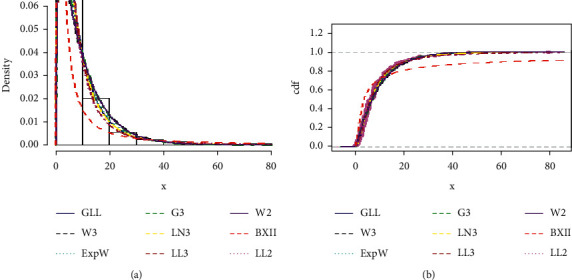
Some estimated fitted densities and cumulative functions of data set I.

The estimated fitted pdfs and CDFs of data set I for the competitive models are shown in Figure 15.

## 7. Bayesian Model Formulation

Given a set of data *x*=(*x*_1_,  *x*_2_,   …,  *x*_*n*_) from GLL (*α*, *k*,  *η*), the likelihood function of the model is given by

(69) $$\begin{array}{c} L\left(\alpha ,\;k,\;\eta |x\right)=\;{\left(\alpha k\;\right)}^{n}\prod_{i=1}^{n}{\left(kx_{i}\right)}^{\alpha -1}\prod_{i=1}^{n}{\left[1+{\left(\eta x_{i}\right)}^{\alpha }\right]}^{-\left(\left(k^{\alpha }/\eta^{\alpha }\right)+1\right)}. \end{array}$$

The Bayesian model is built by specifying the prior distribution for the model parameters *α*, *k* and *η* and then multiplying with the likelihood function *L*(*α*,  *k*,  *η|x*) for the given data *x*=(*x*_1_,  *x*_2_,   …,  *x*_*n*_) to obtain the posterior distribution function using the Bayes theorem. The prior distribution of *α*, *k* and *η* is denoted as *p*(*α*, *k*, *η*).

The joint posterior is

(70) $$\begin{array}{c} p\left(\alpha ,\;k,\;\eta |x\right)\propto L\left(\alpha ,\;k,\;\eta |x\right)p\left(\alpha ,k,\eta \right). \end{array}$$

### 7.1. Prior Distribution

We assumed independent noninformative gamma priors for the parameters of the proposed model in this study due to the flexibility of gamma distributions in accommodating many possible shapes for the types of parameters involved in the proposed distribution. Furthermore, they enable efficient posterior calculations and the recovery of the noninformative distribution for each parameter. Many research papers in the literature consider taking these priors into account (see [28, 37–41]).

For the model parameters, we assume independent gamma priors: *α* ~ *G*(*a*_1_, *b*_1_), *k* ~ *G*(*a*_2_, *b*_2_), and *η* ~ *G*(*a*_3_, *b*_3_).

(71) $$\begin{array}{c} p\left(\alpha \right)=\frac{b_{1}^{a_{1}}}{\Gamma \left(a_{1}\right)}\alpha^{a_{1}-1}\exp\left(-b_{1}\alpha \right),\;\alpha >0,a_{1}>0,b_{1}>0,\\ p\left(k\right)=\frac{b_{2}^{a_{2}}}{\Gamma \left(a_{2}\right)}k^{a_{2}-1}\exp\left(-b_{2}k\right),\;\alpha >0,a_{2}>0,b_{2}>0,\\ p\left(\eta \right)=\frac{b_{3}^{a_{3}}}{\Gamma \left(a_{3}\right)}\eta^{a_{3}-1}\exp\left(-b_{3}\eta \right),\;\eta >0,a_{3}>0,b_{3}>0. \end{array}$$

Hence, we have

(72) $$\begin{array}{c} p\left(\alpha ,k,\;\eta \right)=p\left(\alpha \right)p\left(k\right)p\left(\;\eta \right). \end{array}$$

### 7.2. Posterior Distribution

The posterior expression can be obtained, up to proportionality, by multiplying the likelihood by the prior, and this can be written as

(73) $$\begin{array}{c} p\left(\alpha ,\;k,\;\eta |x\right)\propto \alpha^{a_{1}+n-1}k^{a_{2}+n-1}\eta^{a_{3}+n-1}e^{-\left(b_{1}\alpha +b_{2}k+b_{3}\eta \right)}L_{1}, \end{array}$$

where

(74) $$\begin{array}{c} L_{1}={\left(\alpha k\;\right)}^{n}\prod_{i=1}^{n}{\left(kx_{i}\right)}^{\alpha -1}\prod_{i=1}^{n}{\left[1+{\left(\eta x_{i}\right)}^{\alpha }\right]}^{-\left(\left(k^{\alpha }/\eta^{\alpha }\right)+1\right)}\;. \end{array}$$

The posterior is complicated, and there are no closed-form inferences. As a result, we, propose using McMC techniques to simulate samples from the posterior, allowing for simple sample-based inferences.

### 7.3. Gibbs Sampler: Algorithm

Markov chains require a stationary distribution in order to perform Markov chain Monte Carlo calculations. These chains can be built in a variety of ways. Over the last decade, the following Monte Carlo sampling techniques for assessing high-dimensional posterior integrals have already been developed. Others are Metropolis-Hastings's sampling, Monte Carlo importance sampling, Gibb's sampling, and others. The most popular McMC sampling algorithm in the Bayesian survival inference computation literature is Gibbs' sampling, which is primarily a special case of Metropolis-Hastings's sampling. Gibb's sampling is preferred in high-dimensional numerical computation.

By using Gibbs's sampling, we only need to know the full conditional distribution. To carry out Gibbs's sampling, the basic scheme is as follows:

Step 1: compute the posterior distribution, up to proportionality, and specify the full conditionals, using equation (71), of the model parameters *α*, *η* and *k* as follows.
  (i) Full conditional of *α* given *η*, *k* and *x*:
    (75) $$\begin{array}{c} p\left(\alpha |\eta ,k,x\;\right)\propto \alpha^{a_{1}+n-1}e^{-\left(b_{1}\alpha \right)}L_{1}. \end{array}$$
  (ii) Full conditional of *k* given *α*, *η* and *x*:
    (76) $$\begin{array}{c} p\left(k|\alpha ,\;\eta ,x\right)\propto k^{a_{2}+n-1}e^{-\left(b_{2}k\right)}L_{1}. \end{array}$$
  (iii) Full conditional of *η* given *α*, *k* and *x*:
    (77) $$\begin{array}{c} p\left(\eta |\alpha ,k,x\right)\propto \eta^{a_{3}+n-1}e^{-\left(b_{3}\eta \right)}L_{1}. \end{array}$$
Step 2: select an initial value *θ*^(0)^=(*α*^(0)^, *k*^(0)^,  *η*^(0)^) to start the chain.
Step 3: suppose that, at the *i*th step, *θ*=(*α*, *η*, *k*)  takes the value *θ*^(*i*)^=(*α*^(*i*)^, *k*^(*i*)^,  *η*^(*i*)^); then, from full conditionals, generate
  (78) $$\begin{array}{c} \alpha^{\left(i+1\right)},\text{from }p\left(\alpha |k^{\left(i\right)},\;\eta^{\left(i\right)},x\;\right),\\ k^{\left(i+1\right)},\text{from }p\left(k|\alpha^{\left(i+1\right)},\;\eta^{\left(i\right)},x\;\right),\\ \eta^{\left(i+1\right)},\text{from }p\left(\eta |\alpha^{\left(i+1\right)},\;k^{\left(i+1\right)},x\;\right). \end{array}$$
Step 4: this completes a transition from *θ*^(*i*)^ to *θ*^(*i*+1)^.
Step 5: repeat Step 3 *N* times.

## 8. Bayesian Analysis

In this work, we assumed the independent gamma priors for *α* ~ *G*(*a*_1_, *b*_1_), *k* ~ *G*(*a*_2_, *b*_2_), and *η* ~ *G*(*a*_3_, *b*_3_) with hyperparameter values (*a*_1_=*b*_1_=*a*_2_=*b*_2_=*a*_3_=*b*_3_=1.0).

### 8.1. Convergence Diagnostics

The proposed model is built with the goal of calculating Bayesian estimates for GLL parameters using the McMC method. Due to the Ergodic property of the Markov chain, all inferences are based on the assumption that it will converge. Hence, the McMC convergence diagnostic is crucial. If the simulated sample gives an acceptable approximation for the posterior density, the inferences are correct. Several convergence diagnostic analyses are used to determine whether the chains have converged, including the following.

#### 8.1.1. Geweke's Convergence Diagnostic

**Figure 16:**
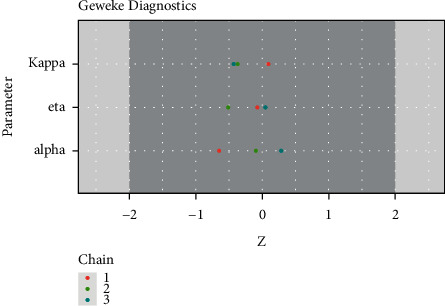
Geweke's diagnostic plot for alpha, eta, and kappa parameters.

Geweke's diagnostic, also called Geweke's *z*-score diagnostic, focuses on comparing the first and last parts of a chain. It is, in fact, a frequentist comparison, of means, with 95 percent of the values falling between −2 and 2, as proposed by [42]. All three values of the three parameters for the three chains in Figure 16 are between −2 and 2.

#### 8.1.2. Autocorrelation Diagnostics

**Figure 17:**
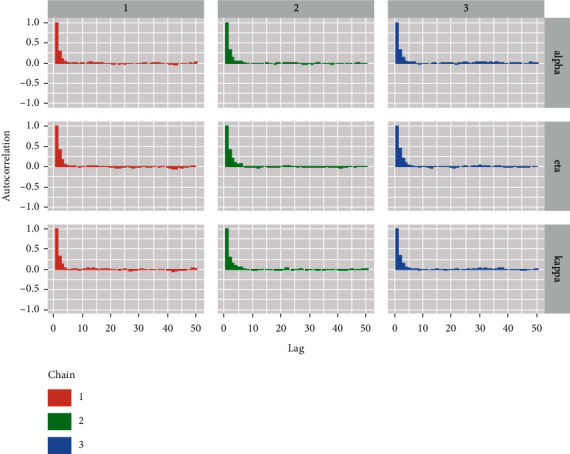
Autocorrelation plot for the alpha, eta, and kappa parameters.

The autocorrelation plot for the parameters is shown in Figure 17.

#### 8.1.3. Heidelberger and Welch's Convergence Diagnostic

Schruben [43] and Schruben et al. [44] proposed detecting nonstationarity in simulation output using a spectral analysis approach to estimate the sample mean variance. They applied the Cramer-von Mises statistic and Brownian bridge theory to test the null hypothesis of stationarity of the Markov chain.

Heidelberger and Welch [45] applied the aforementioned test to introduce a comprehensive method for generating a confidence interval of a predetermined width for the mean of a parameter when the chain has an initial transient (a state when the algorithm has not reached stationarity yet). They computed a test statistic (based on the Cramer-von Mises test statistic) to reject or accept the null hypothesis that the Markov chain belongs to a stationary distribution. A single chain was subjected to diagnostic.

#### 8.1.4. Raftery and Lewis's Diagnostic

Raftery and Lewis [46, 47] proposed “a method for a single chain that tests for chain convergence to the target distribution and estimates the run-lengths required to properly estimate quantiles of functions of the parameters.”

In this study, we applied a quantile of interest (0.025), the desired level of accuracy of ±0.0005, and a probability of 0.95 to attain the indicated degree of accuracy.

#### 8.1.5. Brooks–Gelman–Rubin (BGR) Convergence Diagnostic

**Figure 18:**
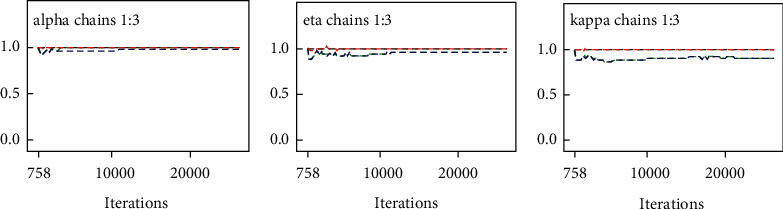
BGR plots for alpha, eta, and kappa parameters.

The fact that the lines for all of the parameters are close to 1 indicates convergence from BGR plots as shown in Figure 18.

In this section, a summary of some common statistical convergence diagnostics tests is provided in Table 10.

#### 8.1.6. Ergodic Mean (Running Mean) Plot

**Figure 19:**
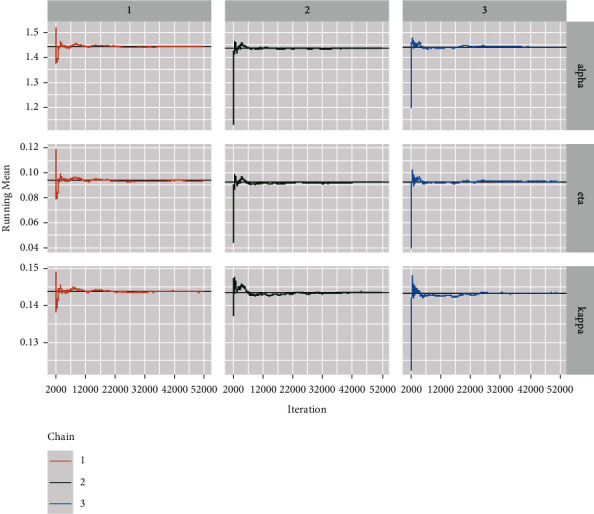
The ergodic mean plots for alpha, eta, and kappa.

The running mean, also known as the ergodic mean, is the average of all samples up to and including a specific iteration. It is used to observe the McMC chains' convergence pattern.  Figure 19 shows a time-series graph of each parameter and it displays the running mean (or ergodic mean) plots for the three parameters of the GLL distribution. The running mean plots of alpha, eta, and kappa show that the chains converge to the values in Table 11 after *N* iterations.

### 8.2. Posterior Analysis

In this section, we present numerical and visual summaries of the posterior distribution for each of the three chains. The joint posterior distribution for the proposed model was estimated using the JAGS software [48]. For each proposed model, we ran three parallel chains with 50,000 iterations and a burn-in of 5,000. Chains were thinned by storing every fifth iteration to reduce autocorrelation in the sample. The use of various convergence diagnostic tools ensured convergence to the joint posterior.

#### 8.2.1. Numerical Summary

We have considered different quantities of interest and their numeric data based on an McMC sample of posterior properties for generalized log-logistic distribution. The McMC simulation results include the results of of the posterior mean, posterior standard deviation, naïve standard error, time-series standard error, Markov chain error, the posterior five-point summary statistics (minimum, lower quartile (Q1), median (Q2), upper quartile (Q3), and maximum), the posterior skewness, posterior kurtosis, 2.5th percentile, 97.5th percentile, and the credible interval followed by the highest probability density (HPD).

The naïve standard error is defined as a measure of simulation error in the mean rather than posterior uncertainty.

(79) $$\begin{array}{c} \text{naive SE}=\;\frac{\text{posterior SD}}{\sqrt{n}}. \end{array}$$

The time-series SE adjusts the “naïve” SE for autocorrelation.

#### 8.2.2. Visual Summary

In this subsection, we have considered different graphs for a visual summary of the posterior properties; those include the box plot, density strip plots, histogram, and trace plots for the parameters. These graphs and plots provide a nearly complete picture of the parameters' posterior uncertainty [49]. We applied the posterior sample (*α*^(*j*)^, *k*^(*j*)^ and *η*^(*j*)^), *j*=1,…, 15000, to draw these graphs.

**Figure 20:**
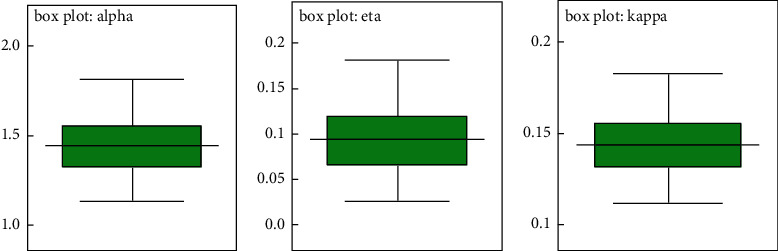
The box plots for the alpha, eta, and kappa parameters.

*(1) Box Plots*. The boxes in Figure 20 represent interquartile ranges, and the line in the middle of each box is the median; the arms of each box extend to encompass the central 95 percent of the distribution, and their ends thus correspond to the 2.5 percent and 97.5 percent quartiles, respectively.

**Figure 21:**
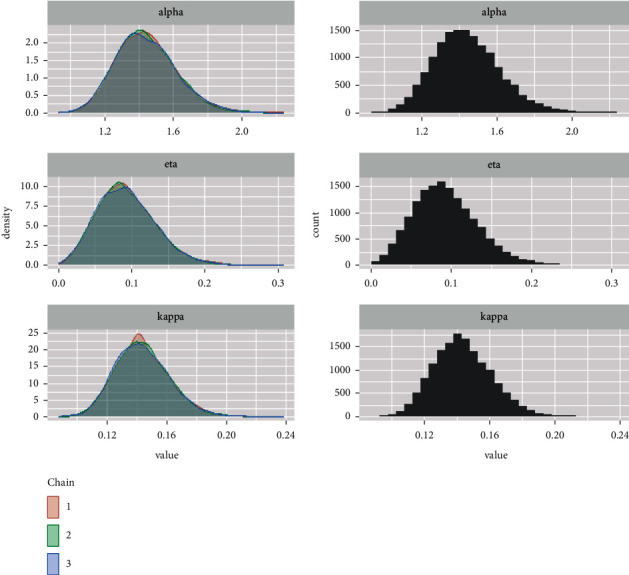
Kernel density estimate and the histogram plots for alpha, eta, and kappa parameters.

**Figure 22:**
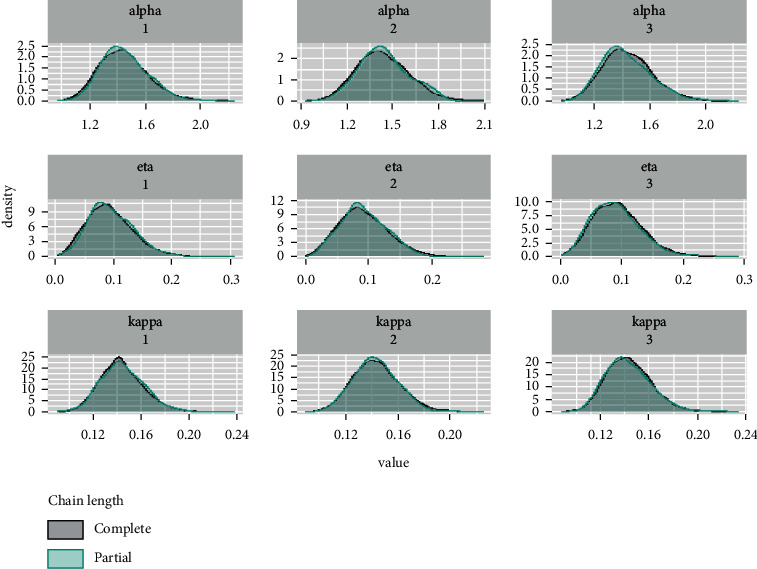
Density plots for the parameters comparing the whole chains with their last parties.

*(2) Density and Histogram Plots*. Histogram can provide information about the behaviour in the tails, skewness, data outliers, and the presence of multimodal behaviour. The graphs in Figure 21 can provide us with a nearly complete picture of the posterior uncertainty about the GLL parameters, while the graphs in Figure 22 show a comparison of the full density and partial density of the parameters.

**Figure 23:**
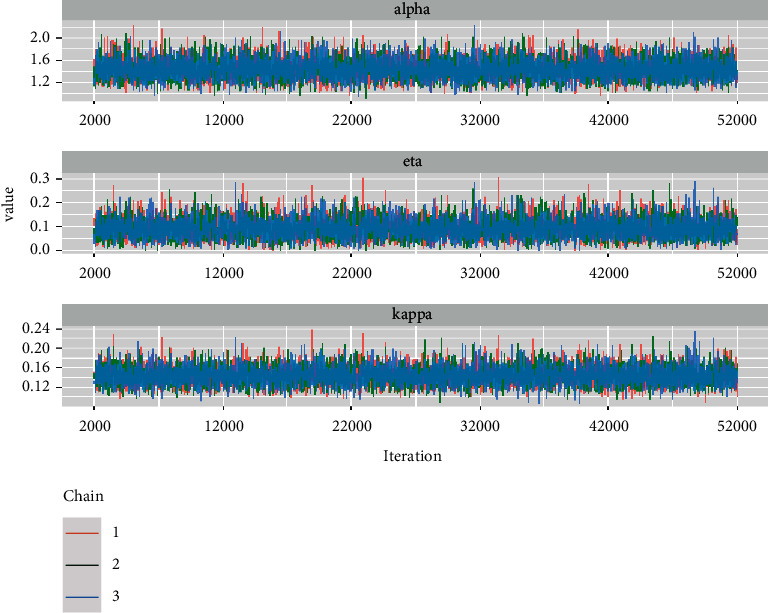
The trace plots for alpha, eta, and kappa parameters.

*(3) Trace Plots*. A trace plot, also known as “a time-series plot,” is a representation of the iteration number versus the value of the parameter drawn at each iteration. Because the plots do not show long-term increasing or decreasing trends but rather resemble a horizontal band in Figure 23, we can conclude that the chains have converged.

## 9. Conclusions

This work introduced and presented results on the mathematical and statistical properties of the generalized log-logistic distribution. The GLL model contains several parametric survival submodels that could be used in a variety of statistics and probability applications. Statistical properties such as quantile function and their related results, moments and their related results, *r*^th^ central moments, and residual and reversed residual life were derived. We have also considered the Bayesian and classical inference of the unknown parameters of the proposed distribution when the data is uncensored or complete. The Bayesian estimates are obtained using the Gibbs sampling method under the assumption of independent gamma priors on the shape and scale parameters. It is worth noting that when prior information is available, Bayes estimates clearly outperform maximum likelihood estimates. To assess the behaviour of the estimators, Monte Carlo simulations are run. The proposed distribution was also applied to a real-world data set and provided a better fit than its submodels and other common parametric survival distributions based on goodness-of-fit statistics, log-likelihood function, and information criterion values. As a result, we conclude that the GLL is the most appropriate model among the distributions considered and it is a very competitive model for explaining lifetime phenomena.

This work has numerous potential extensions. In practice, for example, the presence of explanatory variables and long-term survivals is common. Furthermore, a regression model for both complete and incomplete (or censored) data could be beneficial. As a result, our framework can be further researched in these contexts. The GLL distribution could also be useful in studies comprising survival models such as accelerated failure time, competing risks, mixture cure, frailty, multiple states, and joint survival models, as well as longitudinal data.

## Figures and Tables

**Table 1:** Summary of submodels from the GLL distribution.

Distributions	*α*	*η*	*k*
Log-logistic distribution	*α*	*η*=*k*	*k*=*η*
Weibull distribution	*η* ^*α*^⟶0	*η* ^*α*^⟶0	*k*
Exponential distribution	*α*=1	*η*⟶0	*k*
Standard log-logistic distribution	*α*	*η*=*k*=1	*k*= *η*=1
Burr XII distribution	*α*	*η*=*kλ*^−(1/*α*)^, *λ* > 0	*η*=*kλ*^−(1/*α*)^, *λ* > 0

**Table 2:** Quantiles of the proposed distribution for different parameter values.

Quantiles	(*k*, *α*, *η*)				
	(0.5, 0.5, 0.5)	(5.0, 1.5, 1.5)	(4.0, 4.0, 2.5)	(3.0, 2.0, 3.0)	(5.0, 3.0, 2.0)
0.1	0.0247	0.0449	0.1427	0.1111	0.0945
0.2	0.1250	0.0745	0.1725	0.1667	0.1216
0.3	0.3673	0.1026	0.1945	0.2182	0.1424
0.4	0.8889	0.1314	0.2134	0.2722	0.1608
0.5	1.9999	0.1627	0.2312	0.3333	0.1783
0.6	4.4999	0.1985	0.2489	0.4082	0.1961
0.7	10.8889	0.2421	0.2681	0.5092	0.2155
0.8	32.0000	0.3006	0.2906	0.6667	0.2385
0.9	162.0000	0.3972	0.3222	1.0000	0.2707

**Table 3:** 1st five moments, standard deviation, skewness, and kurtosis of the GLL distribution for some parameter values.

Moments	(*k*, *α*, *η*)						
	(0.5, 0.5, 0.5)	(1.0, 1.5, 1.5)	(1.5, 2.0, 2.5)	(2.0, 5.0, 3.0)	(1.0, 1.0, 2.0)	(4.0, 4.5, 0.2)	(5.0, 4.0, 0.5)
*μ*_1_′	0.1034	0.2065	0.2432	0.2795	0.1547	0.2281	0.1813
*μ*_2_′	0.0567	0.1292	0.1482	0.1741	0.0893	0.0554	0.0354
*μ*_3_′	0.0388	0.0925	0.1036	0.1204	0.0619	0.0141	0.0073
*μ*_4_′	0.0294	0.0715	0.0787	0.0900	0.0471	0.0037	0.0016
*μ*_5_′	0.0237	0.0581	0.0631	0.0711	0.0380	0.0010	0.0004
SD	0.2146	0.2943	0.2984	0.3098	0.2557	0.0575	0.0509
CV	2.0743	1.4250	1.2270	1.1081	1.6529	0.2521	0.2805
CS	2.3743	1.1784	0.9109	0.6100	1.6648	−0.1784	−0.0871
CK	7.8842	3.0318	2.5240	2.0238	4.6628	2.8081	2.7479

**Table 4:** Monte Carlo simulation results for the GLL distribution: MLE, AB, MSEs, and RMSEs.

Parameters	*n*	I			II		
		MLE	AB	RMSE	MLE	AB	RMSE
*α*	50	2.320	1.420	5.273	2.330	1.530	7.149
	100	1.281	0.381	2.386	1.097	0.297	2.263
	300	0.995	0.095	1.369	0.937	0.137	1.512
	600	0.921	0.021	0.5207	0.840	0.040	0.619
	900	0.908	0.008	0.067	0.804	0.004	0.058
	1200	0.905	0.005	0.060	0.803	0.003	0.049
	1500	0.904	0.004	0.054	0.804	0.004	0.045

*k*	50	1.246	0.746	2.306	1.217	0.817	2.802
	100	0.792	0.292	1.235	0.613	0.213	1.093
	300	0.571	0.071	0.665	0.463	0.063	0.467
	600	0.511	0.011	0.135	0.422	0.022	0.199
	900	0.508	0.008	0.095	0.405	0.005	0.081
	1200	0.507	0.007	0.082	0.404	0.004	0.066
	1500	0.505	0.005	0.073	0.405	0.005	0.063

*η*	50	3.280	0.780	3.404	3.033	1.033	3.944
	100	2.780	0.280	1.800	2.281	0.281	1.614
	300	2.612	0.112	0.904	2.056	0.056	0.806
	600	2.554	0.054	0.588	2.046	0.046	0.543
	900	2.542	0.042	0.500	2.019	0.019	0.442
	1200	2.526	0.026	0.409	2.014	0.014	0.360
	1500	2.520	0.020	0.370	2.030	0.030	0.340

**Table 5:** The remission times (in months) of a sample of 128 bladder cancer patients.

3.88, 5.32, 7.39, 10.34, 14.83, 34.26, 0.90, 2.69, 4.18, 5.34, 7.59, 10.66, 15.96, 36.66, 1.05, 2.69, 4.23, 5.41, 7.62, 10.75, 16.62, 43.01, 1.19, 2.75, 4.26, 5.41, 7.63, 17.12, 46.12, 1.26, 2.83, 4.33, 5.49, 7.66, 11.25, 17.14, 79.05, 1.35, 2.87, 5.62, 7.87, 11.64, 17.36, 1.40, 3.02, 4.34, 5.71, 7.93, 0.08, 2.09, 3.48, 4.87, 6.94, 8.66, 13.11, 23.63, 0.20, 2.23, 3.5, 4.98, 6.97, 9.02, 13.29, 0.40, 2.26, 3.57, 5.06, 7.09, 9.22, 13.80, 25.74, 0.50, 2.46, 3.64, 5.09, 7.26, 9.47, 14.24, 25.82, 0.51, 2.54, 3.70, 5.17, 7.28, 9.74, 14.76, 26.31, 0.81, 2.62, 3.82, 5.32, 7.32, 10.06, 14.77, 32.15, 2.64, 11.79, 18.10, 1.46, 4.40, 5.85, 8.26, 11.98, 19.13, 1.76, 3.25, 4.50, 6.25, 8.37, 12.02, 2.02, 3.31, 4.51, 6.54, 8.53, 12.03, 20.28, 2.02, 3.36, 6.76, 12.07, 21.73, 2.00, 3.36, 6.93, 8.65, 12.63, 22.69.

**Table 6:** Descriptive statistics of data set I.

Mean	Median	Mode	Variance	Skewness	Kurtosis	Minimum	Maximum
9.365	6.395	5	110.435	3.286	15.481	0.08	79.05

**Table 7:** Likelihood ratio test statistic for data set I.

Distribution	Hypothesis	LRT	*p* values
W2	*H*_0_ : *η*^*α*^⟶0 vs *H*_1_ : *H*_0_ is false	8.676	0.003
LL2	*H*_0_ : *η*^*α*^=*k* vs *H*_1_ : *H*_0_ is false	10.819	0.001
Burr XII	*H*_0_ : *kλ*^−(1/*α*)^, *λ* > 0 vs *H*_1_ : *H*_0_ is false	87.472	<0.001
Ex	*H*_0_ : *η*=0&*α*=1 vs *H*_1_ : *H*_0_ is false	9.182	0.010
Standard LL	*H*_0_ : *η*=*k*=1 vs *H*_1_ : *H*_0_ is false	190.150	<0.001

**Table 8:** Information criterion for data set I.

Distribution	AIC	BIC	CAIC	HQIC
GLL	**825.564**	**834.120**	**825.756**	**829.040**
LN3	826.723	835.279	826.916	830.199
LL2	826.937	835.641	827.033	829.254
ExpW	827.393	835.949	827.586	830.869
LL3	827.458	836.014	827.651	830.934
G3	831.955	840.511	832.148	835.431
W2	832.163	837.868	832.259	834.481
W3	832.665	841.221	832.858	836.141
Burr XII	910.959	916.663	911.055	913.276

**Table 9:** MLE estimators of the model parameters, the log-likelihood, and goodness-of-fit statistics for data set I.

Distributions	Estimates (SEs)	*ℓ*	*W* ^*∗*^	*A* ^*∗*^	K − S (*p* value)
GLL (*α*, *kη*)	*α* = 1.410 (0.174)	**−409.78**	**0.019**	**0.128**	**0.034** **(0.999)**
	*k* = 0.134 (0.017)				
	*η* = 0.077 (0.038)				

ExpW (*α*, *kλ*)	*α* = 0.275 (0.146)	−410.70	0.045	0.291	0.044 (0.967)
	*k* = 0.676 (0.136)				
	*λ* = 2.636 (1.161)				

LL3 (*α*, *β*, *γ*)	*α* = 0.535 (0.061)	−410.73	0.019	0.135	0.038 (0.993)
	*β* = 1.863 (0.106)				
	*μ* = −0.293 (0.358)				

LN3 (*α*, *β*, *γ*)	*α* = 0.877 (0.090)	−410.36	0.017	0.115	0.029 (0.998)
	*β* = 1.925 (0.111)				
	*μ* = −0.623 (0.372)				

G3 (*α*, *β*, *γ*)	*α* = 1.098 (0.134)	−412.98	0.125	0.778	0.067 (0.618)
	*β* = 8.424 (1.238)				
	*μ* = 0.075 (0.018)				

W3 (*α*, *β*, *γ*)	*α* = 1.031 (0.072)	−413.33	0.134	0.839	0.080 (0.387)
	*β* = 9.743 (0.908)				
	*μ* = 0.077 (0.013)				

W2 (*α*, *β*)	*α* = 1.049 (0.068)	−414.08	0.131	0.784	0.071 (0.545)
	*k* = 9.576 (0.854)				

BXII (*α*, *β*)	*α* = 2.342 (0.356)	−453.48	0.752	4.564	0.251 (<0.005)
	*k* = 0.233 (0.040)				

LL2 (*α*, *β*)	*α* = 0.578 (0.043)	−411.47	0.043	0.310	0.041 (0.984)
	*k* = 1.805 (0.088)				

**Table 10:** Summary of some statistical convergence diagnostic tests.

Parameter	Geweke's diagnostic	Raftery and Lewis	Heidelberger-Welch		
	Pr > |*z*|	Total no. of samp.	*p* value	Stationarity test	Halfwidth test
Alpha	−1.1992	3823	0.072	Passed	Passed
Eta	−0.5711	4338	0.690	Passed	Passed
Kappa	0.4144	4106	0.980	Passed	Passed

**Table 11:** Numerical summaries of posterior properties for the GLL model with gamma priors based on an McMC sample.

Characteristics	Chain 1			Chain 2			Chain 3		
	*α*	*η*	*k*	*α*	*η*	*k*	*α*	*η*	*k*
Mean	1.444	0.094	0.144	1.437	0.093	0.144	1.441	0.093	0.143
SD	0.175	0.041	0.018	0.172	0.040	0.018	0.174	0.040	0.018
Naïve SE	0.002	0.001	0.0002	0.002	0.001	0.0002	0.002	0.001	0.0002
Time-series SE	0.003	0.0003	0.0003	0.003	0.0003	0.0004	0.003	0.001	0.0003
MC error	0.001	0.0004	0.0001	0.001	0.0003	0.0002	0.002	0.002	0.0001
Minimum	0.967	0.003	0.090	0.933	0.001	0.090	0.965	0.001	0.088
2.5th percentile	1.139	0.027	0.112	1.134	0.025	0.112	1.134	0.024	0.112
Q1	1.319	0.065	0.131	1.316	0.064	0.131	1.316	0.064	0.131
Medium (Q2)	1.432	0.090	0.142	1.425	0.088	0.143	1.425	0.090	0.142
Q3	1.550	0.118	0.155	1.548	0.117	0.155	1.553	0.118	0.155
97.5th percentile	1.825	0.184	0.182	1.820	0.180	0.181	1.820	0.180	0.183
Maximum	2.240	0.307	0.238	2.087	0.281	2.392	2.233	0.290	0.233
Mode	1.450	0.090	0.145	1.450	0.090	0.145	1.350	0.090	0.145
Variance	0.031	0.002	0.0003	1.636	0.002	0.0003	0.030	0.002	0.0003
Skewness	0.463	0.655	0.484	0.372	0.514	0.375	0.365	0.524	0.423
Kurtosis	0.372	0.821	0.612	0.153	0.344	0.301	0.044	0.420	0.412
95% credible interval	(1.139, 1.825)	(0.027, 0.184)	(0.112, 0.182)	(1.134, 1.820)	(0.025, 0.180)	(0.112, 0.181)	(1.134, 1.820)	(0.024, 0.180)	(0.112, 0.183)
95% HPD interval	(1.113, 1.784)	(0.021, 0.174)	(0.112, 0.181)	(1.104, 1.764)	(0.021, 0.171)	(0.110, 0.179)	(1.107, 1.779)	(0.019, 0.172)	(0.108, 0.178)

## Data Availability

The data used to support the findings of this study are included within the article.
