# Supplementary material for: Bayesian and Classical Inference for the Generalized Log-Logistic Distribution with Applications to Survival Data
Source: Comput Intell Neurosci. 2021 Oct 11;2021:5820435. doi: 10.1155/2021/5820435 (PMC8523281; doi:10.1155/2021/5820435)
Supplement: Supplementary Materials — The supplementary materials are available in the supplementary file. [file 5820435.f1.docx]

**SUPPLEMENTARY MATERIAL ASSOCIATED FOR THE PAPER**

**####BAYESIAN AND CLASSICAL INFERENCE FOR THE GENERALIZED LOG-LOGISTIC DISTRIBUTION ###**

**######Data Set I**

library(AdequacyModel)

library(rootSolve)

library(FAdist)

x=insert data here

x

#Shape of the Hazard Rate

TTT(x, col = "green", lwd = 2.5, grid = TRUE, lty = 2)

#Generalized LL-pdf distribution function.

pdf_GLL<-function(par,x){

alpha = par[1]

k = par[2]

eta = par[3]

((alpha*k)*((x*k)^(alpha-1)))/(1+((x*eta)^alpha))^(((k^alpha)/(eta^alpha))+1)

}

#Generalized LL-cdf distribution function.

#Generalized LL-Cumulative Distribution Function

cdf_GLL<-function(par,x){

alpha = par[1]

k = par[2]

eta = par[3]

(1-((1+((x*eta)^alpha))^(-((k^alpha)/(eta^alpha)))))

}

set.seed(0)

goodness.fit(pdf=pdf_GLL, cdf=cdf_GLL, starts=c(0.1,0.1,0.1), data= x,

method= "BFGS", domain = c(0,Inf), mle=NULL)

**# Monte-Carlo Simulation Study**

par<-c(0.4,0.8,2.0)

n_replicas=1500

matriz_par<-matrix(0,30,3)

matriz_bias<-matrix(0,30,3)

matriz_MSE<-matrix(0,30,3)

matriz_RMSE<-matrix(0,30,3)

matriz_std<-matrix(0,30,3)

colnames(matriz_par)<-c("kappa","alpha", "eta")

colnames(matriz_bias)<-c("kappa","alpha", "eta")

colnames(matriz_MSE)<-c("kappa","alpha", "eta")

colnames(matriz_RMSE)<-c("kappa","alpha", "eta")

colnames(matriz_std)<-c(("kappa","alpha", "eta")

cont=1

n=50

while(n<=1500){

par_mean<-c(0,0,0)

std_mean<-c(0,0,0)

bias<-c(0,0,0)

MSE<-c(0,0,0)

replica=1

while(replica<=n_replicas)

{

print(paste("n=",n,",replica=",replica))

x<-rGLL(par,n)

Data<-x

kappa=0.4

alpha=0.8

eta=2.0

#############Optimization and Generating the Simulation results#################3

result<-nlminb(c(kappa, alpha,eta), LL_fxn, lower=0, upper = Inf)

if (class(result) !="try-error" && result$convergence==0)

{

par_mean<-par_mean+result$par

bias=bias+(result$par-par)

MSE=MSE+(result$par-par)^2

replica=replica+1

}

}

par_mean=par_mean/n_replicas

bias=bias/n_replicas

MSE=MSE/n_replicas

RMSE=sqrt(MSE)

matriz_par[cont,]=par_mean

matriz_std[cont,]=std_mean

matriz_bias[cont,]=bias

matriz_MSE[cont,]=MSE

matriz_RMSE[cont,]=RMSE

print("mean= ")

print(par_mean)

print("bias= ")

print(bias)

print("MSE= ")

print(MSE)

print("RMSE= ")

print(RMSE)

n=n+50

cont=cont+1

}

print(matriz_par)

print(matriz_MSE)

print(matriz_bias)

print(matriz_RMSE)

n=seq(50,1500,50)

###################################

**############# WinBUGS Code###################################**

**#Model**

model

{

for (i in 1:N)

{

zeros[i] <- 0

phi[i] <- -log(L[i])

zeros[i] ~ dpois(phi[i])

L[i] <- (alpha*kappa*pow((x[i]*kappa),(alpha-1)))/pow((1+pow((x[i]*eta),alpha)),(((pow(kappa,alpha)/pow(eta,alpha))+1)))

}

alpha ~ dgamma(1,1)

kappa ~ dgamma(1,1)

eta~ dgamma(1,1)

}

**#Initial Parameters**

list(alpha=1.5, kappa=1.0, eta=1.0)

list(alpha=2.5, kappa=2.0, eta=1.5)

list(alpha=3.5, kappa=3.0, eta=2.0)

**#Data set**

#DATA SET 3 - Bladder Cancer Patients

list(N=128,

x=insert data here

**############# JAGS Code###################################**

#Model

model{

for (i in 1:N)

{

phi[i] <- -log(L[i])

zeros[i] ~ dpois(phi[i])

L[i]<-(alpha*kappa*pow((st[i]*kappa),(alpha1)))/pow((1+pow((st[i]*eta),alpha)),(((pow(kappa,alpha)/pow(eta,alpha))+1)))

}

#Priors

alpha ~ dgamma(10,10)

kappa ~ dgamma(10,10)

eta~ dgamma(10,10)

}

#Code

###Bayesian Inference of the Generalized Log-logistic Distribution

#DATA SET 3 - Bladder Cancer Patients

x=insert data here

st=x

d.jags<-list(N=128,st=st, zeros=rep(0,128))

i.jags<-function(){list(alpha=runif(0.1), kappa=runif(0.1), eta=runif(0.1))}

p.jags<-c( "alpha","kappa", "eta")

library(rjags)

library(runjags)

library(BRugs)

modelCheck("st.txt")

m1<-jags.model(file="st.txt", data = d.jags, inits = i.jags, n.chains = 3)

update(m1,1000)

res<-coda.samples(m1,variable.names = p.jags, n.iter = 50000, thin = 10)

summary(res)

result<-as.mcmc(do.call(rbind,res))

dic.samples(m1, n.iter = 1000, thin=1, type="pD")
